# Genetic diversity of *Coffea arabica* L. mitochondrial genomes caused by repeat- mediated recombination and RNA editing

**DOI:** 10.3389/fpls.2023.1261012

**Published:** 2023-10-11

**Authors:** Yang Ni, Xinyi Zhang, Jingling Li, Qianqi Lu, Haimei Chen, Binxin Ma, Chang Liu

**Affiliations:** Center for Bioinformatics, Institute of Medicinal Plant Development, Chinese Academy of Medical Sciences, Peking Union Medical College, Beijing, China

**Keywords:** *Coffea arabica*, mitochondrial genome, repetitive sequence analysis, homologous recombination, RNA editing

## Abstract

**Background:**

*Coffea arabica* L. is one of the most important crops widely cultivated in 70 countries across Asia, Africa, and Latin America. Mitochondria are essential organelles that play critical roles in cellular respiration, metabolism, and differentiation. *C. arabica*’s nuclear and chloroplast genomes have been reported. However, its mitochondrial genome remained unreported. Here, we intended to sequence and characterize its mitochondrial genome to maximize the potential of its genomes for evolutionary studies, molecular breeding, and molecular marker developments.

**Results:**

We sequenced the total DNA of *C. arabica* using Illumina and Nanopore platforms. We then assembled the mitochondrial genome with a hybrid strategy using Unicycler software. We found that the mitochondrial genome comprised two circular chromosomes with lengths of 867,678 bp and 153,529 bp, encoding 40 protein-coding genes, 26 tRNA genes, and three rRNA genes. We also detected 270 Simple Sequence Repeats and 34 tandem repeats in the mitochondrial genome. We found 515 high-scoring sequence pairs (HSPs) for a self-to-self similarity comparison using BLASTn. Three HSPs were found to mediate recombination by the mapping of long reads. Furthermore, we predicted 472 using deep-mt with the convolutional neural network model. Then we randomly validated 90 RNA editing events by PCR amplification and Sanger sequencing, with the majority being non-synonymous substitutions and only three being synonymous substitutions. These findings provide valuable insights into the genetic characteristics of the *C. arabica* mitochondrial genome, which can be helpful for future study on coffee breeding and mitochondrial genome evolution.

**Conclusion:**

Our study sheds new light on the evolution of *C. arabica* organelle genomes and their potential use in genetic breeding, providing valuable data for developing molecular markers that can improve crop productivity and quality. Furthermore, the discovery of RNA editing events in the mitochondrial genome of *C. arabica* offers insights into the regulation of gene expression in this species, contributing to a better understanding of coffee genetics and evolution.

## 1 Introduction

*Coffea arabica* L. belongs to the family Rubiaceae (Noir et al., 2004). It was widely distributed in tropical and subtropical regions of Asia, Africa, and Latin America (Ferreira T. et al., 2019). *C. arabica* is an essential agricultural crop mainly grown as a cash crop in tropical countries (Vidal et al., 2010). Caffeine is the main active ingredient of coffee and has many bioactive effects, such as neuroprotection, improving vascular function, reducing blood sugar, and protecting the liver (Cano-Marquina et al., 2013; Bhupathiraju et al., 2014; Kolahdouzan and Hamadeh, 2017). The first draft genome sequence of *C. canephora*, a close relative of *C. arabica*, was assembled to provide insights into the evolution of caffeine biosynthesis (Denoeud et al., 2014). Lashermes et al. studied the molecular characterization and origin of the *C. arabica* genome, revealing specific sequences and conserved regions. Mekbib et al. found that the SNPs might contribute to the genetic variations associated with important agronomic traits such as caffeine content, yield, disease, and pest in *C. arabica* (Mekbib et al., 2022). Min et al. (2019) and Park et al. (2019) reported the chloroplast genome of *C. arabica* and developed the molecular markers. However, the *C. arabica* mitochondrial genome remains unreported.

Mitochondria are semi-autonomous, membrane-bound organelles (Margulis and Bermudes, 1985). According to the endosymbiosis theory, they originated from an engulfed alpha-proteobacterium, which eventually formed a symbiotic relationship with the host cells (Poole and Penny, 2007). Their primary functions involve providing ATP to cells via oxidative phosphorylation (Shtolz and Dan, 2019) and synthesizing metabolic precursors (Scott and Logan, 2007). Moreover, mitochondria play vital roles in cell differentiation, growth, division, and programmed cell death (Zamzami et al., 1997; Valero, 2014).

The mitochondrial genome structure of angiosperms varies significantly among species (Handa, 2003; Sloan et al., 2012). Higher plant mtDNA is abundant in repeat sequences, which mediate homologous recombination and contribute to the evolution of plant mitochondrial genomes (Chevigny et al., 2020). Homologous recombination is essential for maintaining genomic stability, enhancing genomic diversity, driving genomic evolution, and adapting to environmental changes. The location, frequency, and type of recombination can influence genome structure and evolution (Gualberto and Newton, 2017). This widespread homologous recombination results in the complexity of plant mitochondrial genomes (Chevigny et al., 2020).

Earlier studies have demonstrated that mtDNA exhibits intricate and dynamic structures, including linear and branched chromosomes, which may be intermediates in replication or recombination and represent multiple genome isoforms (Backert et al., 1997). In some cases, mtDNAs were found as a master circular molecule (Cheng et al., 2021; Fang et al., 2021; Ni et al., 2022), such as in *Panax ginseng* (Jang et al., 2021) and *Vitex rotundifolia* (Bendich, 1996). In the *Coriandrum sativum*, the mitochondrial genomes consist of two circular molecules (lengths 82,926 bp and 224,590 bp) (Kozik et al., 2019), while experiments by Kozik et al. revealed that the predominant form of mitochondrial DNA molecules in Lactuca sativa (Kozik et al., 2019) is simple and branched linear.

RNA editing refers to the phenomenon that the base changes occur at the molecular level of the mRNA produced by transcription, including the insertion, deletion, and replacement of nucleotides and other different ways, resulting in its sequence cannot complement the gene coding sequence and the amino acid composition of the protein produced by translation also changes, which is a supplement to the central dogma (Gott and Emeson, 2000). Three study groups first documented RNA editing in flowering plant mitochondria 30 years ago (Covello and Gray, 1989; Araya et al., 1995). It was soon discovered that RNA editing in plants also occurs in chloroplasts (Hoch et al., 1991), but there are generally fewer RNA base changes in angiosperms than in mitochondria. Comparison of organelle RNA sequences with their corresponding mtDNA sites shows that editing events most often occur at the first or second codon position and may affect the amino acids defined by the mitochondrial genome (Lenz et al., 2018a). Many of these nucleotide changes lead to codon changes that specify amino acids highly conserved in evolution (Mower, 2005; Sloan, 2017; Edera et al., 2018; Lenz et al., 2018b; Brenner et al., 2019). RNA editing predominantly affects non-synonymous positions of protein-coding regions (Picardi et al., 2010; Grewe et al., 2014; Grimes et al., 2014), changing the resulting amino-acid sequences (Gualberto et al., 1989).

The Rubiaceae family, a diverse and significant group in the plant kingdom, has garnered attention for its mitochondrial genome variations and peculiarities. *Damnacanthus indicus* showcases dynamic evolution with unique gene and intron content, serving as the first reference mitochondrial genome for the Rubiaceae family (Han et al., 2021). The *Neolamarckia cadamba* mitochondrial genome not only revealed its phylogenetic position within the Gentianales order but also shed light on its taxonomic relationship within the Rubiaceae family (Wang et al., 2021). Oldenlandia corymbosa’s smaller mitochondrial genome size compared to other family members indicates variations in size and content within the Rubiaceae species (Julca et al., 2023). The intricacies of the *Psychotria viridis* mitochondrial genome, presented through multiple mitogenome structures and evidence of heteroplasmy, enrich our understanding of mitochondrial genome organization (Varani et al., 2022). Meanwhile, the mitogenome of *Scyphiphora hydrophyllacea* stands out due to its intricate intron content and its phylogenetic positioning within the Gentianales (Chen Y. et al., 2020). These studies collectively offer invaluable insights into the mitochondrial genomes of Rubiaceae species, furthering the understanding of their evolution and diversity.

Here, we first sequenced the complete *C. arabica* mitochondrial genome. The coffee mitochondrial genome contained multiple conformations resulting from recombination mediated with repetitive elements. In addition, we identified 54 fragments that were likely to originate from the chloroplast genome. Lastly, we identified 90 RNA editing sites. These results demonstrated multiple mechanisms that led to the diversity of *C. arabica* mitochondrial genome. This study provides a theoretical basis for the evolution of *C. arabica* organelle genomes and molecular breeding.

## 2 Materials and methods

### 2.1 Plant materials, DNA extraction, and sequencing

We collected *C. arabica* plants from the Shunlong Nursery Farm, located in Baojingyuan Village, Hongyang Town, Puning City, Jieyang City, Guangdong Province, China. The geographic coordinates are Longitude: 116.25001 and Latitude: 23.43801. The altitude of the collection site is 55 meters above sea level. The soil at the collection site of *C. arabica* plants is classified as “Wuni Ditian”, or “Black Clay Paddy Soil”, primarily found in the regions of Guangdong province such as Huiyang, Zhanjiang, Maoming, Zhaoqing, Guangzhou, and Shaoguan (http://vdb3.soil.csdb.cn/) and cleaned the fresh leaves with DPEC water. For NGS sequencing, we extracted DNA using the Magnetic Plant Genomic DNA Kit (Tiangen, China) and constructed a short-read DNA library with an insert size of 350 bp using the TIANSeq Fast DNA Library Kit (catalog number NG102, Illumina, California, USA). We sequenced this library on an Illumina HiSeq X sequencer (Illumina, USA).

For long-read sequencing using Oxford Nanopore technology, we extracted DNA with the NEB Monarch HMW DNA Extraction Kit (catalog number: T3060L, New England Biolabs, Massachusetts, USA). We then constructed a DNA library with 10 kb fragment insert sizes using the DNA Library Kit (catalog number: SQK-LSK110, TIANGEN) and sequenced it on a PromethION sequencer (Novogene Co., Ltd., Beijing, China).

### 2.2 Organelle genome assembly and annotation

The organelle genomes were assembled using a hybrid assembly strategy (**Figure S1**). In step 1, we extracted the cpgenome reads with the parameters “-R 15 -k 21,45,65,85,105 -F embplant_pt” and the mitochondrial genome reads with the parameters “-R 20 -k 21,45,65,85,105 -P 1000000 -F embplant_mt” using GetOrganelle software (Jin et al., 2020) from Illumina data (SRA Accession Number: SRR17345023). In step 2, the short reads were *de novo* assembled using the SPAdes software (Bankevich et al., 2012) embedded in Unicycler software (Wick et al., 2017) into a unitig graph. In step 3, the double bifurcating structures (DBS) in the unitig graph were resolved by mapping the Nanopore long reads (SRA Accession Number: SRR17345007) using Unicycler software (Wick et al., 2017). The hybrid assembly strategy could minimize the false assemblies generated in the polishing step resulting from the interference from Nuclear Mitochondrial DNAs (NUMTs) and Mitochondrial Plastid DNAs (MTPTs) sequences (Timmis et al., 2004; Hazkani-Covo et al., 2010).

We annotated the cpgenome using the CPGAVAS2 web server with database 2 (Shi et al., 2019), and the annotation was checked by the CPGView web server (Liu S. et al., 2023). The *C. arabica* mitochondrial genome was annotated with the Geseq web server (Michael et al., 2017) and IPMGA webserver (http://www.1kmpg.cn/mga/). The mitochondrial genome annotation results were visualized using the OGdraw web server (Stephan et al., 2019). The annotation errors were manually corrected with Apollo software (Lewis et al., 2002).

### 2.3 Tandem repeat elements analysis

We identified two kinds of tandem repeat elements. The Simple Sequence Repeat (SSRs) were detected using the Misa web server (Beier et al., 2017) with default parameters. The tandem repeats were identified using the TRF (Tandem repeats finder) webserver with default parameters (Benson, 1999). The distribution of these repeat elements was visualized by the Circos package (Zhang et al., 2013) embedded in the TBtools (Chen C. et al., 2020).

### 2.4 Identification and validation of repeats able to mediate homologous recombination

We used bioinformatic analysis and experimental methods to verify the possible presence of repeat-mediated recombination products of the mitochondrial genome. For the bioinformatics method, we mapped the long reads (Nanopore data of WGS) to the sequences corresponding to the hypothetical recombination products. We first identified the high-scoring sequence pairs (HSPs) using the BLASTn program (Chen et al., 2015). It should be pointed out that the sequences from a HSP can be considered a pair of dispersed repeat units with low level of sequence similarity. Then, we extracted 1,000 bp long flanking sequences on both sides of each HSP sequence. The resulting two sequences corresponded to two conformations (c1 and c2). Then we recombined the two sequences in silico to create sequences corresponding to the two recombined conformations (c3 and c4). We mapped the Nanopore reads to the sequences corresponding to c1-4. If there were long reads spanned the repeat region of a conformation, we considered the corresponding conformation present.

The presence of recombination products around these repeats was further validated using the PCR amplification and Sanger sequencing methods. The IDT SciTools (Owczarzy et al., 2008) was used for designing the primers specific to amplify each conformation using PCR. The primer sequences are listed in **Table S1**. We used approximately 1 μl DNA, 1 ul 10 μ M each of the forward and reverse primer, 13 μl 2× Taq PCR Master Mix, and 10 μl ddH2O for PCR with the following conditions: 94°C for 3 min; 35 cycles of 94°C for 30 s, 60°C for 30 s and 72°C for 1 min; 72°C for 10 min. Lastly, the PCR products with the expected size were further sequenced using the Sanger method.

### 2.5 Identification of mitochondrial plastid DNAs and phylogenetic analysis

To identify potential MTPTs, we conducted a reciprocal BLASTn search between the complete plastome (OL789882) and mitochondrial genomes (OL789880-OL789881). The objective was to discern segments with sequence similarity. In the initial analysis, the entire plastome served as the “query” sequence, while the mitochondrial genome, comprising both chromosome sequences, acted as the “subject” sequence. For the reciprocal analysis, we reversed these roles, using the mitochondrial genomes as the “query” and the plastome as the “subject”. Results from both searches were then combined. The specific parameters we employed for this BLASTn search were “-evalue 1e-5 -outfmt 6” (Chen et al., 2015). The mapping results of reads to the MTPT regions were visualized using Tablet software (Milne et al., 2012) and examined manually.

We used 18 mitochondrial genomes for phylogenetic analysis and selected the two Lamiaceae species *Scutellaria barbata* (NC_065025.1) and *Scutellaria franchetiana* (NC_065026.1) as outgroups. All mitochondrial genomes were downloaded from the RefSeq database with the following accession numbers: *C. arabica* (OL789880.1, OL789881.1), *N. cadamba* (MT320890.1-MT364442.1), *P. serpens* (NC_069806.1), *P. viridis* (NC_066984.1), *S. hydrophyllacea* (NC_057654.1), *D. indicus* (MZ285075.1), *O. corymbose* (OX459128.1), *Gentiana crassicaulis* (OM320814.1), *Gentiana straminea* (OM328072.1), *Hoya lithophytica* (MW719051.1), *Rhazya stricta* (NC_024293.1), *Asclepias syriaca* (NC_022796.1), *Trachelospermum jasminoides* (OR333986.1), *Cynanchum wilfordii* (MH931257.1-MH931259.1) *Cynanchum auriculatum* (MH410146.1-MH410148.1), *Gelsemium elegans* (MN388837.1). The CDS sequences were extracted from Genbank format files using the PhyloSuite software (Zhang et al., 2020) and aligned with MAFFT software (Katoh and Standley, 2013). The aligned sequences were used to construct the phylogenetic tree using IQTREE2 (Hoang et al., 2017) with the maximum-likelihood method. Subsequently, the bootstrap analysis was evaluated using UFBoot with 1,000 replicates (Hoang et al., 2017). Finally, the phylogenetic tree was visualized using the iTOL website (Letunic and Bork, 2019).

### 2.6 Collinearity and gene content analysis

We conducted a collinearity analysis on the mitochondrial genomes of closely related species of *C. arabica* within the Rubiaceae family, as identified in the phylogenetic analysis. Initially, we downloaded the reference genomes from NCBI for this analysis. Subsequently, we employed the online version of MAFFT at https://mafft.cbrc.jp/alignment/server/ for sequence alignment and Dotplot visualization (Katoh and Standley, 2013). Considering the potential annotation errors in the record, we re-annotated all the genome files using IPMGA webserver to initiating the gene statistical analysis. Following this, we utilized a custom script to tally the annotation results.

### 2.7 RNA editing sites analysis

We used the Deepred-mt software to predict RNA editing events using the convolutional neural network (CNN) model (Edera et al., 2021). We retained predictions with probability values greater than 0.9. Subsequently, we randomly validated the prediction results of RNA editing events with PCR amplification and sanger sequencing. The PCGs were amplified with cDNA (complementary DNA) and gDNA (genomic DNA) to identify those RNA editing sites. The protein-coding genes (PCGs) were extracted from Genbank format files using the PhyloSuite software (Zhang et al., 2020). The IDT web server (Owczarzy et al., 2008) was used for designing the PCR primer of all PCGs. The primers used to validate the RNA editing sites are shown in **Table S2**. The PCR experiment conditions were the same as those described in sub-section 2.4. The PCR products were sequenced using the Sanger method and mapped to the protein-coding sequences to validate the RNA editing sites.

## 3 Results

### 3.1 Mitochondrial genome assembly and annotation

**Figure 1:**
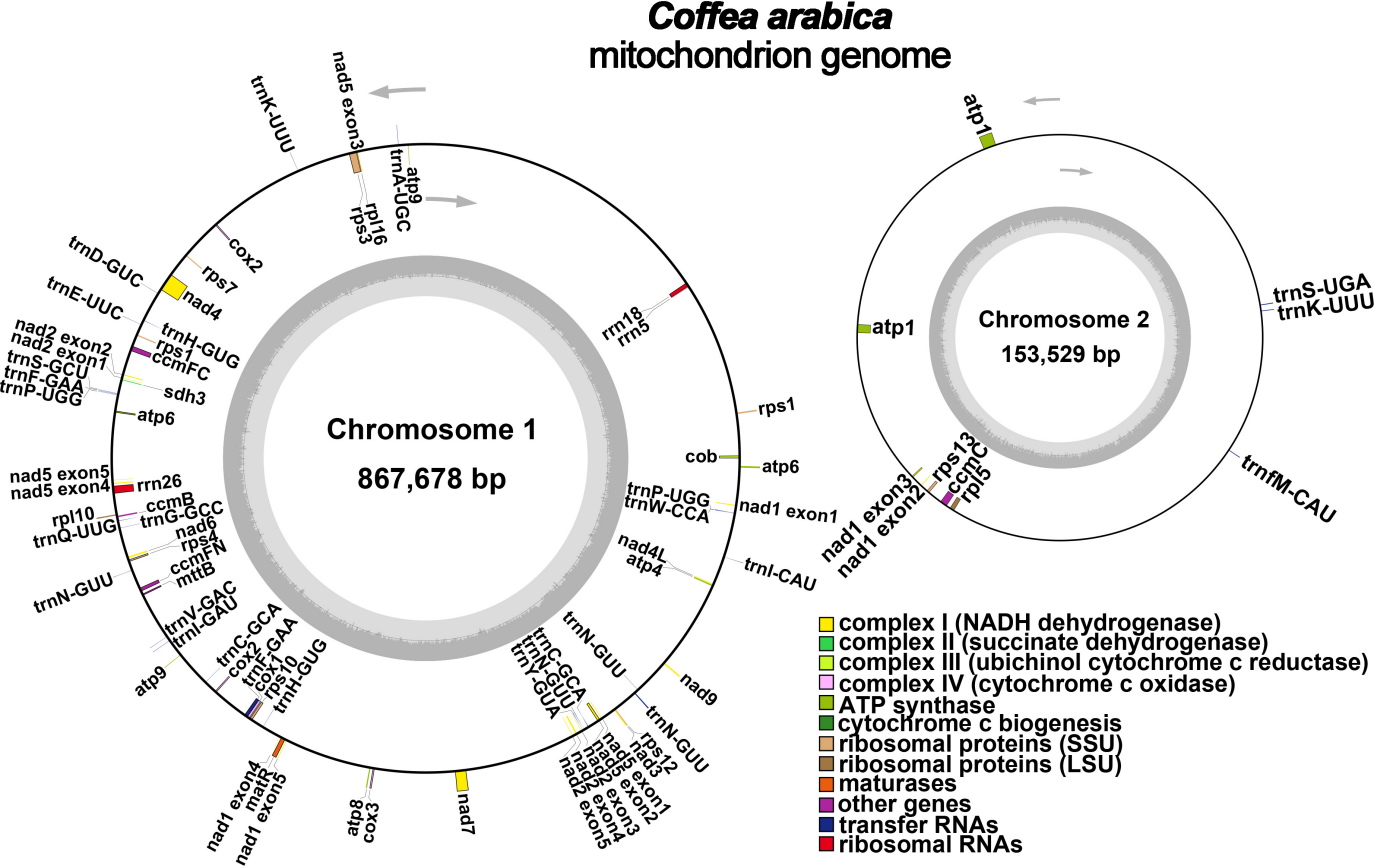
A schematic representation of the *C. arabica mitochondrial genome*. The gene distribution of *C. arabica mitochondrial genome* was shown in different colors based on their functional classification.

We generated 11.1 Gb Nanopore sequencing data (SRA Accession Number: SRR17345007) and 6 Gb Illumina sequencing data (SRA Accession Number: SRR17345023) in total. The mitochondrial genome reads extracted from Illumina data were assembled using SPAdes (Bankevich et al., 2012; Jin et al., 2020) into a unitig graph. The unitig graph contained 13 double-bifurcating structures (DBS) (**Figure S2**). We mapped the long Nanopore reads to each DBS to resolve these DBS using the Unicycler software (**Figure S2**). We defined the major conformations as the two reverse-complementing conformations mapped with more long reads. After retaining only the major conformation, the final assembly contained two mitochondrial genome chromosomes (MC): MC1 and MC2, which were 867,678 bp and 153,529 bp, respectively (**Figure 1**). The total GC content of the *C. arabica* mitochondrial genome MC1 and MC2 was 44.6% and 44.7%, respectively. To compare the mitochondrial genomes of *C. arabica* with those of other Rutaceae species, we analyzed the basic information of 14 released mitochondrial genomes in the NCBI database of Rutaceae, which included *C. arabica* (**Table 1**). Among them, *Neolamarckia cadamba*, which has two chromosomes like *C. arabica*, has a much smaller mitochondrial genome (Total length: 414,980 bp). The *C. arabica* mitochondrial genome is the largest among the 13 species, 104,888 bp larger than the second largest, *Psychotria serpens* (NC_069806.1/MT528155.1). The significant difference in mitochondrial genome size could be attributed to extensive rearrangements and sequence migration during the evolution of the Rutaceae mitochondrial genome (Kitazaki and Kubo, 2010).

**Table 1:** Basic information on mitochondrial genomes of Rubiaceae.

Genome Number	Species	Chromosome Number	Accession Number	Length	Shape	GC content
1	*Coffea arabica*	chromosome 1	OL789880.1	867,678 bp	Circular	44.6%
	*Coffea arabica*	chromosome 2	OL789881.1	153,529 bp	Circular	44.7%
2	*Damnacanthus indicus* var. indicus	NA	MZ285071.1	417,815 bp	Circular	44.5%
3	*Damnacanthus indicus* var. indicus	NA	MZ285072.1	419,010 bp	Circular	44.5%
4	*Damnacanthus indicus* var. indicus	NA	MZ285073.1	419,435 bp	Circular	44.5%
5	*Damnacanthus indicus* var. indicus	NA	MZ285074.1	419,429 bp	Circular	44.5%
6	*Damnacanthus indicus* var. indicus	NA	MZ285075.1	417,661 bp	Circular	44.5%
7	*Damnacanthus indicus* var. indicus	NA	MZ285076.1	417,816 bp	Circular	44.5%
8	*Neolamarckia cadamba*	chromosome 1	MT320890.1	109,836 bp	Circular	45.5%
	*Neolamarckia cadamba*	chromosome 2	MT364442.1	305,144 bp	Linear	45%
9	*Psychotria serpens*	NA	NC_069806.1	916,319 bp	Circular	44.2%
11	*Psychotria viridis*	NA	NC_066984.1	615,370 bp	Circular	44.4%
12	*Psychotria viridis*	NA	ON064100.1	570,344 bp	Circular	44.4%
13	*Scyphiphora hydrophyllacea*	NA	NC_057654.1	354,155 bp	Circular	44.4%
14	*Oldenlandia corymbosa*	NA	OX459128.1	258,274 bp	Circular	44%

All the 24 core PCGs of the plant mitochondrial genome have been found in the mitochondrial genome (**Figure 1**; **Table 2**). These included five ATP synthase genes (*atp1*, *atp4*, *atp6*, *atp8*, and *atp9*); nine NADH dehydrogenase genes (*nad1*, *nad2*, *nad3*, *nad4*, *nad4L*, *nad5*, *nad6*, *nad7*, and *nad9*), four cytochrome c biogenesis genes (*ccmB*, *ccmC*, *ccmFc, ccmFn*), three cytochrome C oxidase genes (*cox1*, *cox2*, and *cox3*), one protein transport subunit (*mttB*), one maturase (*matR)* and one cytochrome oxidase (*cob*). The core genes *atp1*, *atp6*, and *atp9* had two copies in the mitochondrial genome. Besides, there were 12 variable genes in the mitochondrial genome, including three genes encoding the ribosomal protein large subunit genes (*rpl5*, *rpl10*, and *rpl16*), seven genes encoding the ribosomal protein small subunit genes (*rps1*, *rps3*, *rps4*, *rps7*, *rps10, rps12*, and *rps13*), and one succinate dehydrogenase gene (*sdh3, sdh4*). The variable genes *rps1* and *rps12* had two copies in the mitochondrial genome. In addition, we identified three rRNA genes and 26 tRNA genes, corresponding to 18 unique genes, in the *C. arabica* mitochondrial genome.

**Table 2:** Genome composition in the *C. arabica* mitochondrial genome.

Group of genes	Name of genes
ATP Synthase	*atp1* (x2)*, atp4, atp6* (x2)*, atp8, atp9* (x2)
Cytochrome c Biogenesis	*ccmB, ccmC, ccmFc, ccmFn*
Cytochrome b	*cob*
Cytochrome Oxidase	*cox1, cox2, cox3*
Maturase	*matR*
Protein Transport Subunit	*mttB*
NADH Dehydrogenase	*nad1, nad2, nad3, nad4, nad4L, nad5, nad6, nad7, nad9*
Ribosomal Protein Large Subunit	*rpl5, rpl10, rpl16*
Ribosomal Protein Small Subunit	*rps1* (x2)*, rps3, rps4, rps7, rps10, rps12*(x2)*, rps13*
Succinate Dehydrogenase	*sdh3, sdh4*
Ribosomal RNA	*rrn5, rrn18, rrn26*
Transfer RNA	*trn*C-GCA (x2)*, trn*D-GUC*, trn*E-UUC*, trn*F-GAA (x2)*, trn*fM-CAU*, trn*G-GCC*, trn*H-GUG (x2)*, trn*I-GAU*, trn*I-CAU, *trn*K-UUU (x2), *trn*N-GUU (x4)*, trn*P-UGG (x2)*, trn*Q-UUG*, trn*S-GCU*, trn*S-UGA*, trn*V-GAC*, trn*W-CCA*, trn*Y-GUA

### 3.2 Tandem repeat elements analysis

Simple sequence repeats (SSR), or microsatellite sequences, were tandem repeats with shorter repeat units than six bp (Bhattarai et al., 2021). In this study, we used the Misa web server (Beier et al., 2017) to detect the SSRs in the *C. arabica mitochondrial genome.* We identified 270 SSRs in total (**Table S3**). MC1 and MC2 contained 206 and 64 SSRs, respectively. The most abundant type of SSRs were the tetramer SSRs, accounting for 37.41% of the total SSRs. The pentamer and hexameric SSRs were only found in MC1 (**Table S3**).

For the long tandem repeat sequences, twenty-eight were identified by TRF (Benson, 1999) in MC1, and six were found in MC2 (**Table S4**). The tandem repeat with a length of 9 bp from 553,613 to 553,656 had the largest copy numbers (4.9 times).

### 3.3 Dispersed repeat analysis and repeat mediated recombination analysis

A previous study has shown that dispersed repeats can mediate homologous recombination (Li et al., 2021). These dispersed repeats can vary significantly in terms of the percentage of sequence identity. As a result, we used BLASTn to compare the mitochondrial genome sequences to themselves. The resulting similar sequences were called High-scoring sequence pairs (HSP). They are equivalent to the dispersed repeats and were named “R,” followed by their HSP numbers.

We found 515 HSPs in MC1 (OL789880) and MC2 (OL789881) (**Table S5**). We then compared the DBS sequences with these HSP sequences. We found all thirteen DBS sequences identical to some HSP sequences (**Table S5**). We then mapped the long reads to the four conformations of these 13 DBS/HSP. However, the mapping results supported the recombined conformation for only eight HSP (**Table S6**). Among them, six HSPs: R1 (DBS06), R22, R30 (DBS09), R175, R278, and R285, were found in MC1 of *C. arabica*, and the R406, R462 were found shared between the MC1 and MC2 (**Table S6**).

We defined the recombination frequency (RF) as the percentage of reads mapped to the minor conformations divided by those mapped to all four conformations. The RFs for all HSPs were less than 0.22, except for R1, which was 0.54. The HSPs could have contributed to the complex structure of plant mitochondrial genomes and increased the diversity of plant mitochondrial genomes (André et al., 1992).

**Figure 2:**
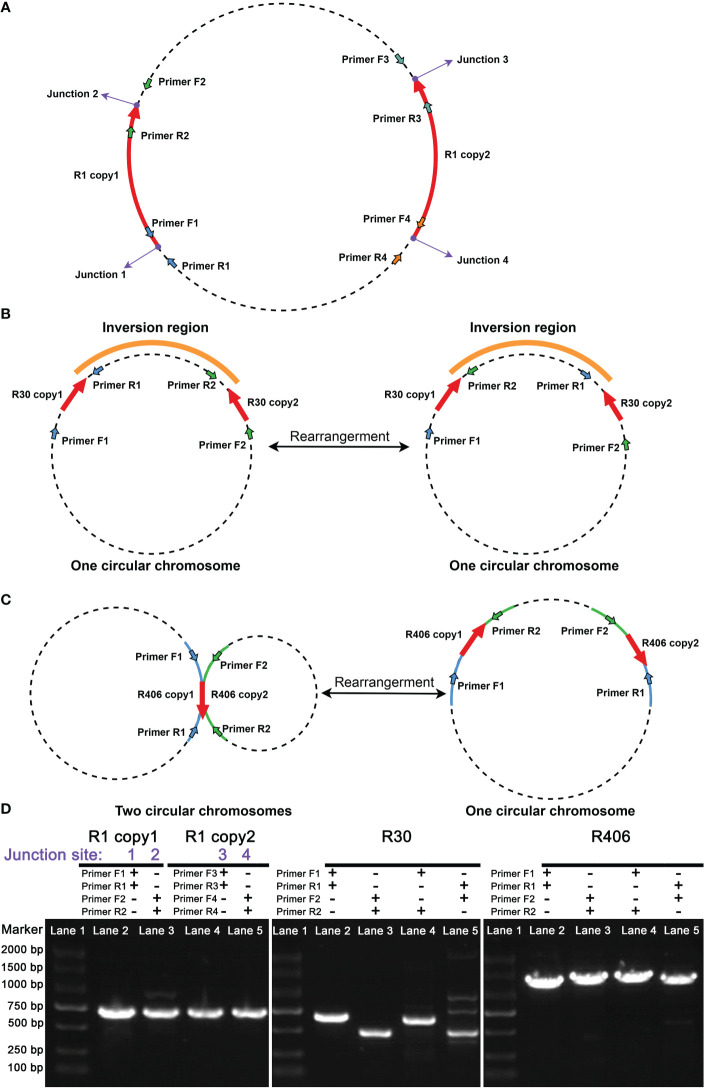
Validation of the repeat mediated recombination products in *C arabica mitochondrial genome*. The purple dots represent junction sites. The red arrows represent the intervals and directions of the repeated sequences. Arrows of other colors represent primer positions. The orange line segment represents the interval that can be inverted. **(A)** The primers were designed for the junction site validation of the R1. Since the repeat sequence, R1, is longer than 5000 bp, it was difficult to obtain PCR products spanning the entire repetitive sequence. Therefore, we only performed PCR to verify the boundaries of R1. **(B)** The primers were designed to validate the major and minor conformations of R30. **(C)** The primers were designed for the major and minor conformations of R406. **(D)** The electrophoresis results of the PCR products for the fragments associated with R1, R30, and R406. The lane numbers and PCR primer names are shown above each panel.

In addition to bioinformatics analysis methods, we used PCR amplification and Sanger sequencing methods to verify the presence of the recombination products corresponding to the minor conformations. We obtained PCR products for those associated with repeats R1, R30, and R406. It should be pointed out that the R1 and R30 corresponded to the bs01 and bs09 found in the unitig graph. The schematic representation of the location of the primers used to amplify the fragments associated with R1 and R30 is shown in **Figures 2A, B**. The electrophoretic results of these recombination products are shown in **Figure 2C**.

**Figure 3:**
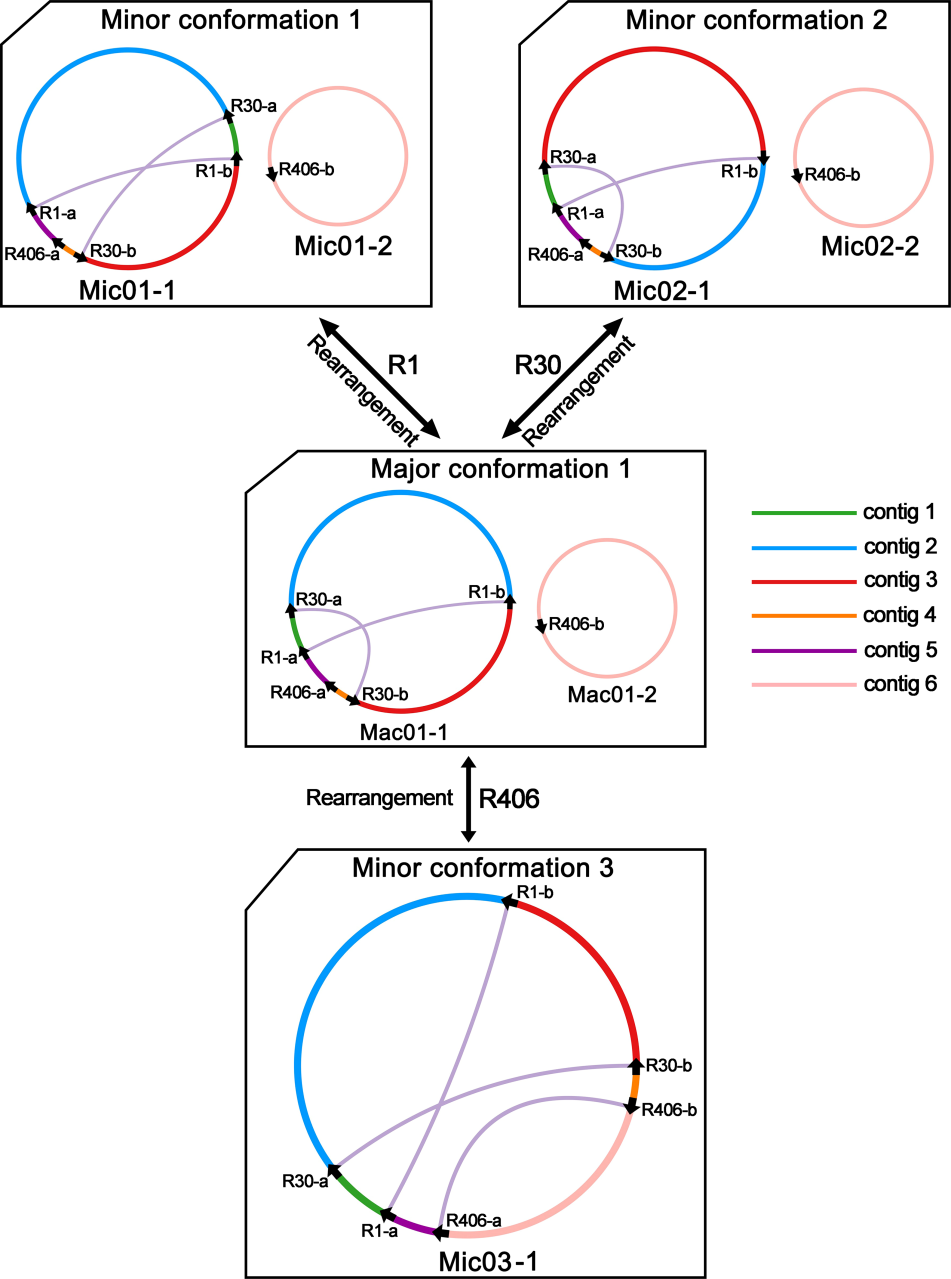
Hypothetical products generated by recombination mediated by repeats R1, R30, and R406 on the chromosomes of *C. arabica mitochondrial genome*. The repeats mediate the structure of the hypothetical recombination products. The circles represent the mitochondrial genome, and the different colored lines represent the regions between the repeated sequences. The black arrows represent the repeat sequence. The original electropherogram of the PCR amplification can be found in **Supplementary File 5**.

In **Figure 2C**, we obtained the PCR products for R1, R30, and R406. The PCR products were sequenced with the Sanger methods. And the results are shown in **Supplementary Files 1–3**. Three pairs of repetitive sequences divided the genome into six contigs (contigs 1-6). Recombination mediated by R1 could rearrange contigs 1 and 2 to form the minor conformation Mic01 (**Figure 3**; **Supplementary File 1**). Similarly, the recombination of R30 could cause the rearrangement of contigs 2 and 3 to form the minor conformation Mic02 (**Figure 3**; **Supplementary File 2**). In contrast, MC1 and MC2 were combined by the recombination-mediated with the direct repeat R406 to form the minor conformation Mic03 (**Figure 3**; **Supplementary File 3**).

### 3.4 Identification of foreign DNAs in the mitochondrial genome

The plant mitochondrial genome could incorporate chloroplast and nuclear DNA during its evolution (Kang et al., 2021). There were 32 and 10 homologous DNA fragments in MC1 and MC2 (**Figure 4**; **Table S7**). The total length was 26,096 bp, 2.55% of the whole mitochondrial genome. The longest fragment was 4,117 bp in MC1, and the shortest was only 31 bp in MC2. We annotated those DNA fragments, and ten complete genes (*rps*7, *psa*A, *trn*N-GUU, *trn*P-UGG, *trn*W-CCA, *trn*D-GUC, *trn*H-GUG, *trn*N-GUU, *trn*I-CAU) were found in those DNA fragments (**Table S7**). All the MTPTs were visualized and checked manually. The presence of long reads spanning the MTPTs supports that these sequences were indeed in the mitochondrial genome (**Figures S3-44**).

**Figure 4:**
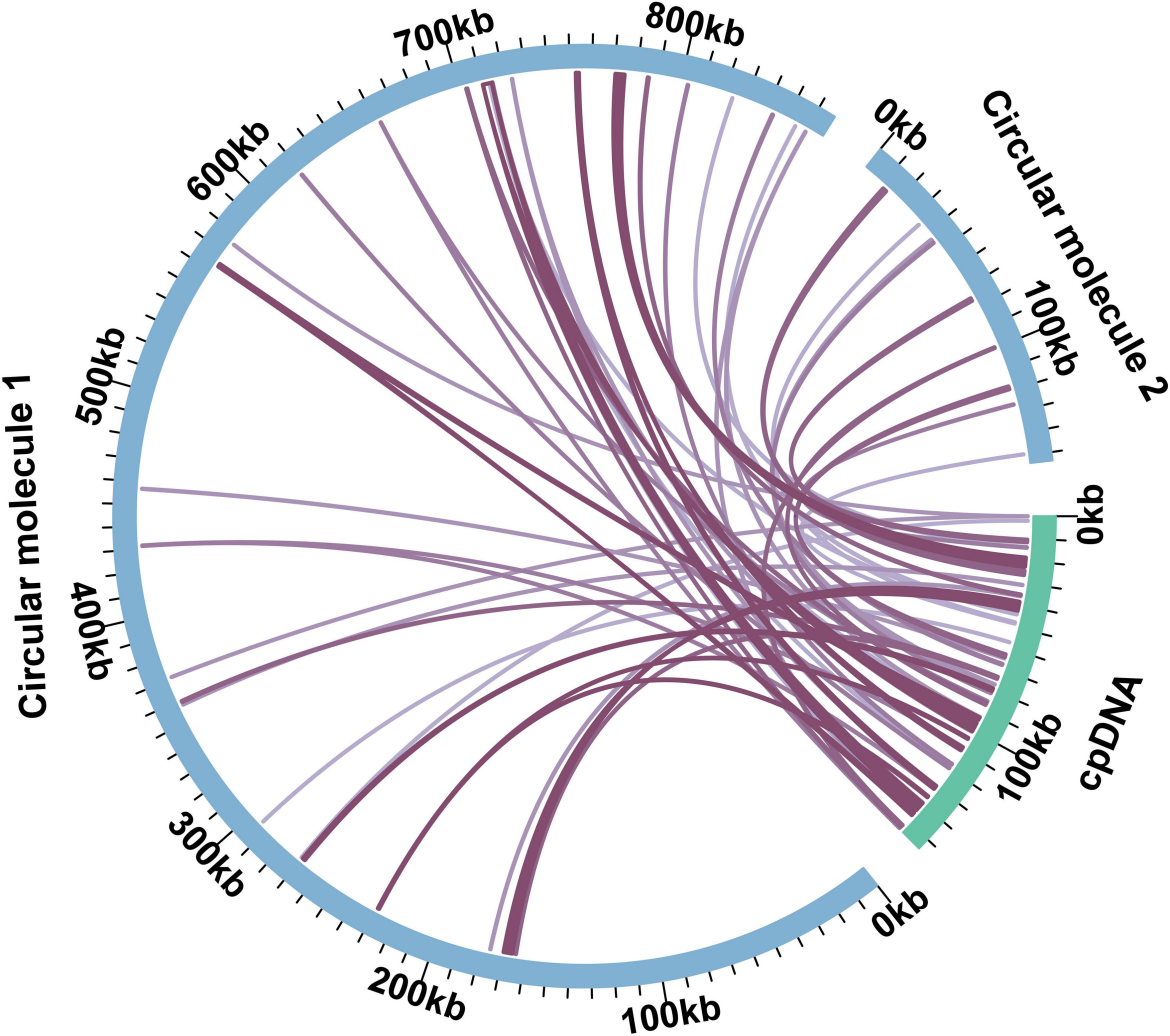
The DNA transfer between the *C. arabica* mitochondrial genome and plastome. The blue arcs represent the mitochondrial genome, and the green arcs represent the chloroplast genome. The purple link in the middle connects the homologous regions between the mitochondrial genome and the plastome.

### 3.5 Phylogenetic analysis

**Figure 5:**
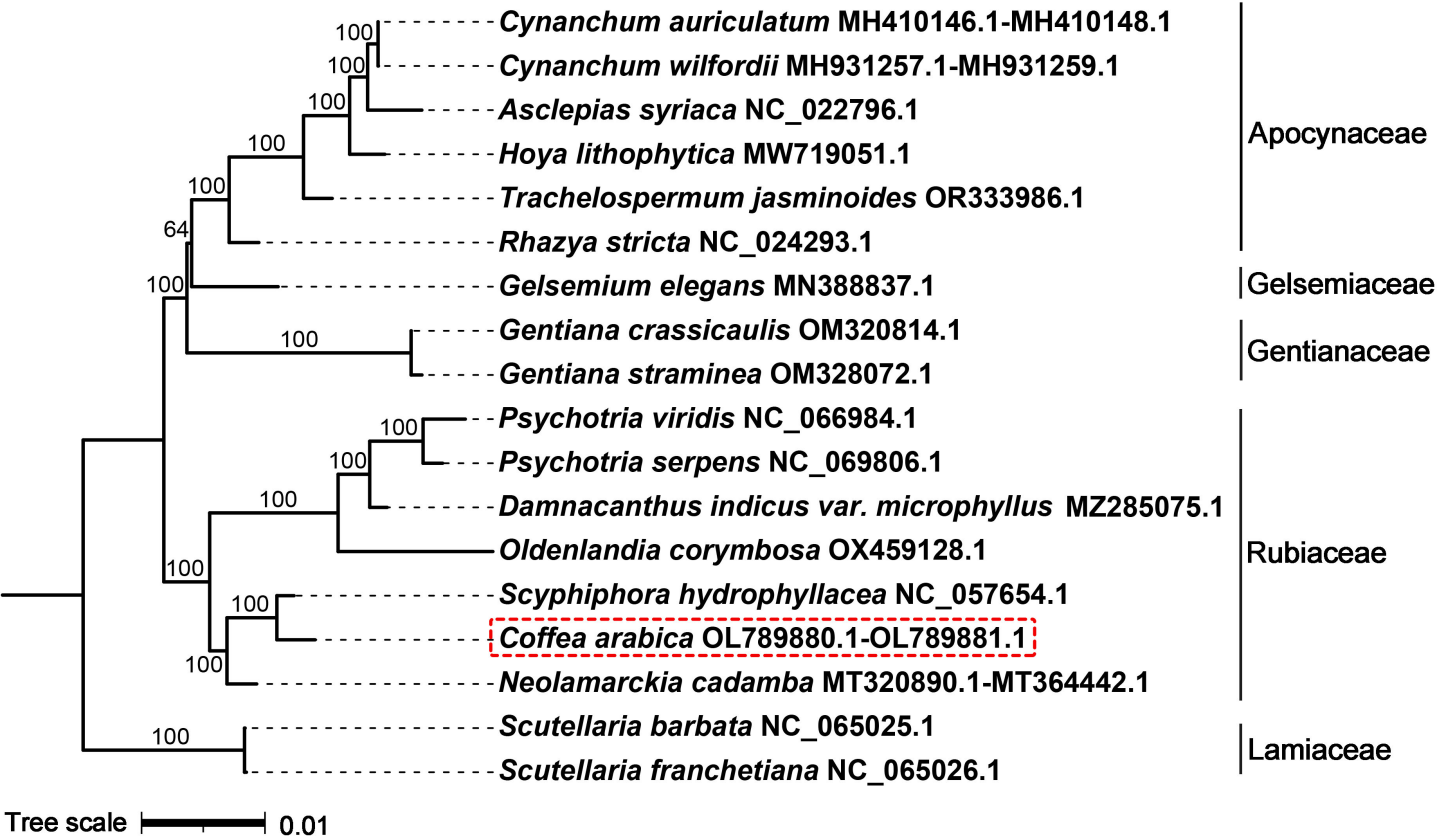
Phylogenetic relationships of *C. arabica* and other 17 species. The black branches represent the phylogenetic relationships constructed by the shared mitochondrial genes. The number on each branch node represents the bootstrap number. The mitochondrial genome NCBI accession number for each species follows the Latin name. The families of these species are shown on the right side.

We conducted the phylogenetic analysis of the mitochondrial genomes of the Rubiaceae, Gentianaceae, Apocynaceae, Gelsemiaceae and Lamiaceae species. thirty-eight common PCGs were identified from these genomes, namely, *atp1, atp4, atp6, atp8, atp9, ccmB, ccmC, ccmFC, ccmFN, cob, cox2, cox3, matR, mttB, nad1, nad2, nad3, nad4, nad4L, nad5, nad6, nad7, nad9, rpl2, rpl5, rpl10, rpl16, rps1, rps2, rps3, rps4, rps7, rps10, rps12, rps13, rps14, rps19* and *sdh3.* The best model was GTR+F+R2, according to the BIC. The phylogenetic tree showed that *C. arabica* and *S. hydrophyllacea* were clustered together with 100% bootstrap values in the context of the currently available mitochondrial genome (**Figure 5**).

### 3.6 Comparative mitogenomic analysis in the Rubiaceae family

By analyzing the protein-coding gene count of the Rubiaceae family, we observed that eight mitochondrial genomes all possess 24 core genes (**Table S8**). Yet, when it comes to variable genes, the protein-coding genes are not conserved. For instance, *C. arabica*, *S. hydrophyllacea*, and *N. cadamba* have one copy of the *sdh3* gene, with *S. hydrophyllacea* containing two copies, while other species lack the *sdh3* gene. *P. viridis* has lost the *rps1* gene, and both *P. viridis* and *P. serpens* are missing the *rps4* gene. Hence, in the Rubiaceae family, although core genes remain relatively conserved, there is a marked disparity in variable genes across different species.

Utilizing collinearity analyses, we observed a non-conserved mitochondrial genome structure within the Rubiaceae family, as evidenced by the limited number of conserved blocks presented in **Figure S45**. Intra-genomic comparisons of *C. arabica* highlighted the presence of a 7804 bp reverse repeat block. Contrastingly, when juxtaposed with other species, with the sole exception of *O. corymbosa*, *C. arabica* exhibited a reverse repeat block spanning an approximate length of 6000-7000 bp. Notably, *O. corymbosa* mitogeome uniquely presented a collinear block around 3500 bp. Such observations underscored the substantial structural diversifications the mitochondrial genomes within the Rubiaceae family have encountered throughout evolutionary processes. It is plausible that these genomic rearrangements are tethered to distinct environmental acclimations, intricate biological interplays, or speciation events.

### 3.7 RNA editing site identification

Using the Deepred-mt program, 472 RNA editing events were predicted (**Table S9**). Of these 472 predicted RNA editing events, the *nad4* gene, *ccmB* gene and *ccmC* gene were edited most frequently, with 36, 34 and 33 editing site respectively. The *sdh3* gene was edited the least for only once (**Figure S46**). To further explore the amino acid changes before and after editing, we counted the amino acid composition before and after RNA editing. Of these 472 RNA editing events, mainly non-synonymous substitutions occurred for 447 times, and synonymous substitutions occurred for a total of 25 times. The most abundant amino acid conversion was Ser to Leu, with a total of 110 times. In addition to this, we found that the *atp6* (CAA to UAA) and the *atp9* genes (CGA to UGA) gained a stop codon through the RNA editing event (**Figure S47**).

**Figure 6:**
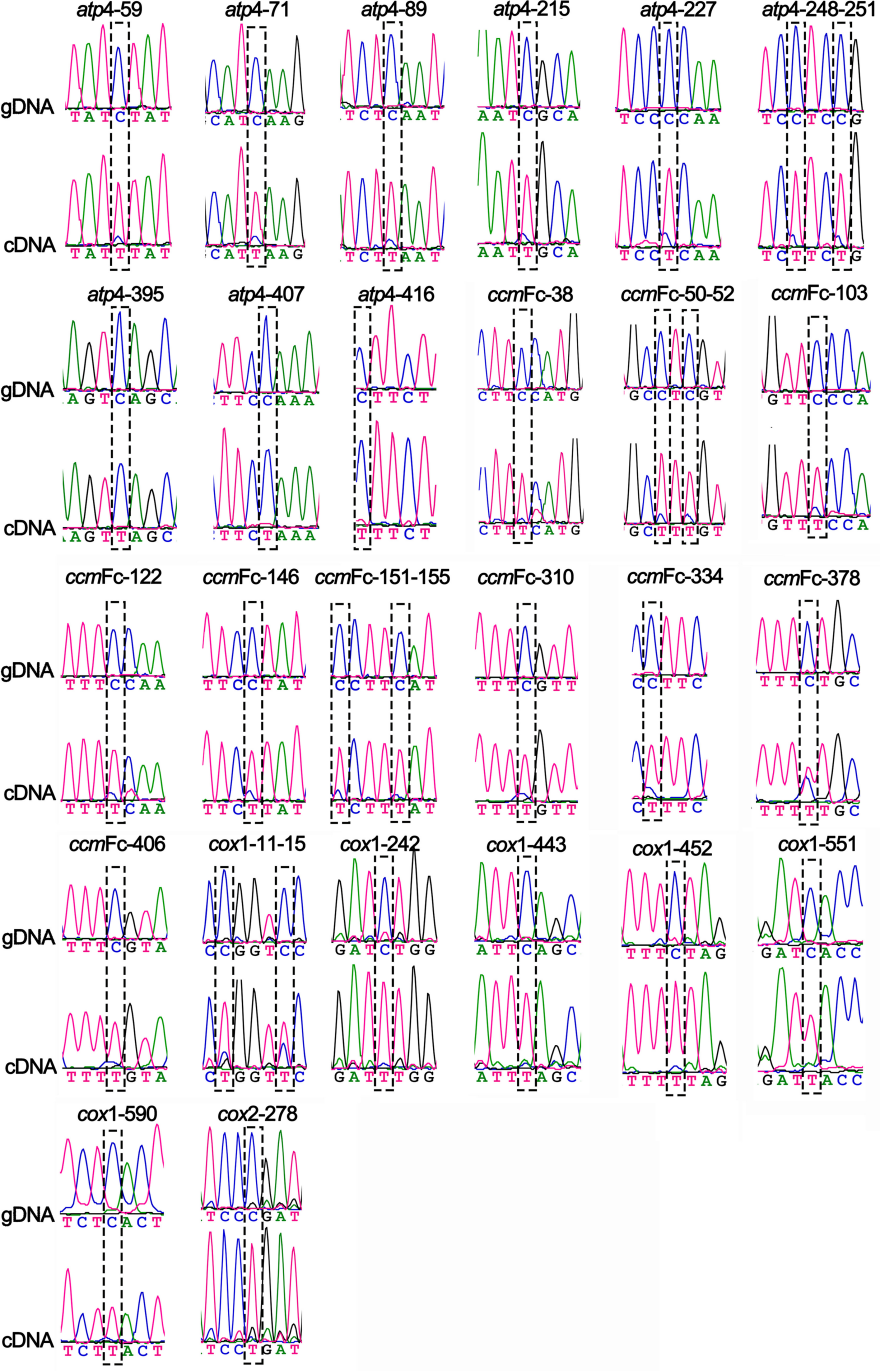
Validation of RNA editing sites in the *atp*4, *ccm*Fc, *cox*1, and *cox*2 genes of *C. arabica mitochondrial genome*. Chromatograms showing the sequences before and after editing at the hypothetical RNA-editing sites. For each RNA editing site, the name is shown on the top. The results from the genomic DNA (gDNA) and complementary DNA (cDNA) are shown in the middle and bottom. Black dashed rectangles framed the RNA editing sites.

To validate that these predicted RNA editing sites were real, we used PCR to amplify all the PCGs of the coffee mitochondrial genome from genomic DNA and cDNA. The products were then sequenced using the Sanger method. A comparison of the results helped us to identify the RNA editing sites. The primers used for the RNA editing site validation can be found in **Table S2**. Finally, we obtained 103 sanger sequencing results of short DNA fragments for the 21 PCGs. We identified 90 RNA-edited events in 14 PCGs. All RNA editing events were of “C to U” and “G to A” types (**Figure 6**; **Figures S48, 49**). The “G to A” type is a symmetric type of the “C to U” type, as discussed before (Wu et al., 2017).

Among the 90 RNA editing events, 87 were non-synonymous substitutions, and only three (*ccm*FC-378, *nad*2-252, and *nad*7-531) were synonymous substitutions. Among the synonymous substitution, the *ccm*FC-378 and *nad*2-252 edited the codon from UUC (F) to UUU (F), and *nad*7-531 edited the codon from UCC (S) to UCU (S). Most of the resulting amino acid changes were from Ser to Leu, which occurred 25 times. The *mtt*B and *nad*2 genes had the most edited sites, including fourteen (**Table S10**). All the RNA editing sites of *ccm*Fc were in the first exon. The *cox*2 gene had only one RNA editing site: *cox*2-278. All the Sanger sequencing results of gDNA and cDNA can be found in the **Supplementary File 4**. The chromatograms of the 90 RNA-editing sites are shown in **Figure 6** and **Figures S48, 49**. Interestingly, all these 87 validated sites were successfully predicted by the Deep-mt program. The three sites with synonymous substitutions were not successfully predicted by the Deep-mt program.

## 4 Discussion

### 4.1 Mitochondrial genome assembly is the current bottleneck in plant mitochondrial genome study

Studies on plant mitochondrial genomes lags behind that of cpgenomes due to the relatively complex structures of plant mitochondrial genomes (Gualberto et al., 2014; Skippington et al., 2015; Skippington et al., 2017). It has been shown that the plant mitochondrial genomes might contain multiple-circular, linear, and branched chromosomes (Kozik et al., 2019; Li et al., 2021; Yang et al., 2022). These intricate structures contribute to challenges in assembling plant genomes.

Two assembly strategies have been proposed for mitochondrial genomes assembly. The first one is similar to the nuclear genome assembly strategy, which uses long reads for *de novo* assembly and short reads for polishing the assembled results (Yu et al., 2011). However, this approach has two major limitations: (1) it cannot restore the complex structure of the mitochondrial genome and often yields a simplified assembly result, such as a single circular molecule (Liu J. et al., 2023); (2) it can introduce false positive results due to the presence of homologous DNA fragments shared between the mitochondrial genomes and the nuclear genomes and the plastomes, such as NUMTs and MTPTs. For example, the reads derived from plastid DNA fragments homologous to MTPT sequences might be used to polish the MTPT sequences, mistakenly changing the MTPT sequences (Yang H. et al., 2022; Jiang et al., 2023; Yang et al., 2023).

A second strategy, which is called the hybrid strategy, has been developed to overcome the above limitations. This strategy assembled the short reads into a unitig graph with different kmer lengths (Jin et al., 2020). Then, the long reads were used to resolve the double bifurcating structures in the unitig graph caused by repetitive sequences in the genome (Park et al., 2018; Wang et al., 2018; Yang et al., 2018; Kim et al., 2020). In our study, we obtained the short mitochondrial genome reads by employing the seed reads extension method through the GetOrganelle software. We utilized this hybrid assembly approach to assemble the *C. arabica* mitochondrial genome, which we discovered contains two chromosomes. We advocate using hybrid strategies to unravel the major conformations of complex plant mitochondrial genome structures.

### 4.2 The complex structure and representation of the plant mitochondrial genomes

Previous studies have shown that the mitochondrial genomes might have circular, linear and multi-branch structures (Gualberto et al., 2014; Chevigny et al., 2020). We found that the coffee mitochondrial genomes can be represented as a unitig graph and two independent chromosomes after splitting the unitig graph based on the mapping results of long reads. However, representing the complex structures of the mitochondrial genomes is a challenging problem. There are two primary methods for addressing this issue.

The first method uses a graph to represent the intricate structure of plant mitochondrial genomes (graph-based method). After assembling the long reads into a graph, the mitochondrial genome structure is represented as multiple connected contigs (Li et al., 2022). This approach has been employed in numerous studies. For example, the mitochondrial genomes of the three species, *Selaginella nipponica*, *Abelmoschus esculentus*, and *Picea sitchensis*, all use this format to represent their mitochondrial genomes and annotate the mitochondrial genomes according to the corresponding mitochondrial contigs (Jackman et al., 2020; Kang et al., 2020; Li et al., 2022).

The second method follows the convention of using circular or linear conformations to represent the mitochondrial genomes (conformation-based method). This approach is based on the understanding that a genome may consist of multiple conformations. Long reads can resolve the unitig graph, resulting in conformations that can undergo homologous recombination mediated by repetitive sequences (André et al., 1992; Alverson et al., 2011). This process can lead to various alternative genomes conformations, typically of low-frequency and referred to as minor conformations. For example, in the *Prunus salicina* mitochondrial genome (Fang et al., 2021), nine repeat sequences were involved in homologous recombination, resulting in two low-frequency chromosomes. Similar findings were observed in the mitochondrial genomes of *Ipomoea batatas* (Yang Z. et al., 2022) and *Scutellaria tsinyunensis* (Li et al., 2021), where direct repeats led to the division of the mitochondrial genome into smaller chromosomes corresponding to minor conformations. In the *Cannabissativa* mitochondrial genome (Liu J. et al., 2023), the major conformation is a circular molecule. However, it can form multiple minor conformations through recombinations mediated by 15 repeat sequences. *Salvia officinalis* has three repeat sequences in the genus *Salvia* (Yang et al., 2023), which can mediate homologous recombination, while there are nine pairs of such repeat sequences in *S. miltiorrhiza* (Yang H. et al., 2022). *Taraxacum mongolicum* has five pairs of repeat sequences confirmed to mediate homologous recombination, producing multiple minor conformations (Jiang et al., 2023).

Both methods have some limitations. Firstly, there are several issues with the graph-based representation of the mitochondrial genome. Until now, most analytic methods and tools for genomes were developed based on the assumption that the genome is a simple circular or linear molecule. Very few methods and tools can be used to analyze a genome represented by a graph (Kang et al., 2020). Secondly, the graph representations of the plant mitochondrial genomes are mostly generated from bioinformatics analysis (Kang et al., 2020). Although the repeat sequences involved could be validated by PCR amplification and Sanger sequencing (Lai et al., 2022), the complete graphic structures of the plant mitochondrial genomes are difficult to validate. Lastly, there is a lack of comparative tools to compare genomes represented as graph. As a result, it would be difficult to conduct comparative genomic studies if the genomes were represented with graph.

Secondly, there are several issues with the conformation-based representation of the mitochondrial genome. Firstly, when resolving the unitig graph, the most abundant conformation was selected at each DBS point. However, whether or not the most abundant conformation forms a chromosome remains to be confirmed. Secondly, the dependence among different repetitive sequences for recombination is not clear. At present time, the minor conformations were generating based on the assumption that the repeat-mediated recombinations are independent of each other. As a result, the exact set of conformation cannot be determined. As a result, the representation of the complex mitochondrial genome structures requires further investigation (de Abreu et al., 2023).

### 4.3 The RNA editing sites “C” to “U.”

RNA editing is a widely observed phenomenon in plant mitochondria (Varré et al., 2019). This process involves modifying the information in transcripts of nearly all angiosperm mitochondrial protein-coding genes (Mower and Palmer, 2006). In our study, we first identified the RNA editing sites using a convoluted neural network-based method: deepred-mt. we validated 90 RNA editing events in the coffee mitochondrial genome, which involved the conversion of cytidine (C) to uridine (U). We had not been able to obtain unambiguous sequencing results for the other nine unigenes after multiple attempts. The likely reasons are that the amplification of the sequences is interfered with highly homologous sequenes such as MTPT or NUMT and etc.

Studies on the model plant *Arabidopsisthaliana* have reported more than 400 RNA editing events involving the substitution of cytidines with uridines in the mitochondrial genome (Bentolila et al., 2013). In *T. mongolicum*, a total of 278 RNA-editing sites were predicted and 213 were validated (Jiang et al., 2023). In *C. sativa* mitochondrion, RNA editing was found to be tissue specific (Liu J. et al., 2023). Using PCR amplification and Sanger sequencing methods, 113 of the 126 RNA editing sites from 11 PCGs were validated in the *S. officinalis*. In the *S. miltiorrhiza*, 225 “C to U” sites in the protein coding regions were discovered (Wu et al., 2017). In the present study, we predicted 472 RNA editing sites, all of which were edited from C to U, which is consistent with the number and type of RNA editing times previously reported.

During plant mitochondrial RNA editing, non-synonymous and synonymous substitutions have different biological significance and impact (Lu et al., 1998). Non-synonymous substitutions involve changes in the amino acid sequence and, therefore, may affect the structure and function of the protein. These changes may result in enhanced, diminished, or complete loss of protein function (Yates and Sternberg, 2013). Of the 472 predicted sites, 447 were non-synonymous. Of the validated 90 sites, 87 were non-synonymous. Only *nad7*-531, *ccmFC*-378, and *nad2*-252 were synonymous. Notably, we found that the stop codons of *atp6* and *atp9* genes were created by RNA editing events from the prediction results of Deepred-mt program. Previous study reported the *atp6* gene premature termination expression in maize, sorghum and *Oenothera* (Kumar and Levings, 1993). The transcripts of *atp9* gene have a stop codon create by the RNA editing events in rapeseed (Handa, 1993). There may be the same possibility of that the same type of (non-synonymous substitution) editing occurred in the coffee. The function of these non-synonymous substitutions, if there are any, still needs to be further investigated.

## 5 Conclusions

In this study, we sequenced and analyzed the *C. arabica* mitochondrial genome. We identified 40 PCGs, 3 rRNA genes, 26 tRNA genes, 270 SSRs, and 34 tandem repeats. In particular, we found three repeats mediating recombination, 54 fragments originating from the chloroplast genome, and 90 RNA-editing sites. We showed that the *C. arabica* mitochondrial genome had a complex structure caused by a plethora of molecular mechanisms.

## Data availability statement

The organelle sequences supporting the conclusions of this article are available in GenBank (https://www.ncbi.nlm.nih.gov/) with accession numbers: OL789880-OL789881 (mitochondrial genome), OL789882 (cpgenome), respectively. The sample was deposited in the Institute of Medicinal Plant Development (Beijing, China) with accession numbers 1KMG_CF01. The raw data has been released through GenBank with the following accession numbers: (1) Nanopore DNA reads BioProject PRJNA792668, BioSample SAMN24449604, SRA database SRR17345007. (2) Illumina DNA reads: BioProject PRJNA792714, BioSample SAMN24451482, SRA database SRR17345023.

## Ethics statement

We collected fresh leaf materials of *Coffea arabica* for this study. The Institute of Medicinal Plants identified the plant sample with Yang Ni. We prepared and deposited the voucher specimens in the Institute of Medicinal Plant Development (Beijing, China) with accession numbers 1KMG_CF01. The study, including plant sample collection, complies with relevant institutional, national, and international guidelines and legislation. No specific permits are required for plant collection.

## Author contributions

CL: Conceptualization, Data curation, Formal Analysis, Funding acquisition, Investigation, Methodology, Project administration, Resources, Software, Supervision, Visualization, Writing – review & editing. YN: Conceptualization, Data curation, Formal Analysis, Investigation, Methodology, Project administration, Software, Supervision, Validation, Visualization, Writing – original draft, Writing – review & editing. XZ: Formal Analysis, Resources, Software, Validation, Writing – review & editing. JL: Formal Analysis, Investigation, Methodology, Software, Writing – review & editing. QL: Formal Analysis, Methodology, Validation, Visualization, Writing – review & editing. HC: Data curation, Formal Analysis, Supervision, Validation, Writing – review & editing. BM: Formal Analysis, Validation, Writing – review & editing.
