# Supplementary figures and images for: Genetic diversity of Coffea arabica L. mitochondrial genomes caused by repeat- mediated recombination and RNA editing
Source: Front Plant Sci. 2023 Oct 11;14:1261012. doi: 10.3389/fpls.2023.1261012 (PMC10598636; doi:10.3389/fpls.2023.1261012)

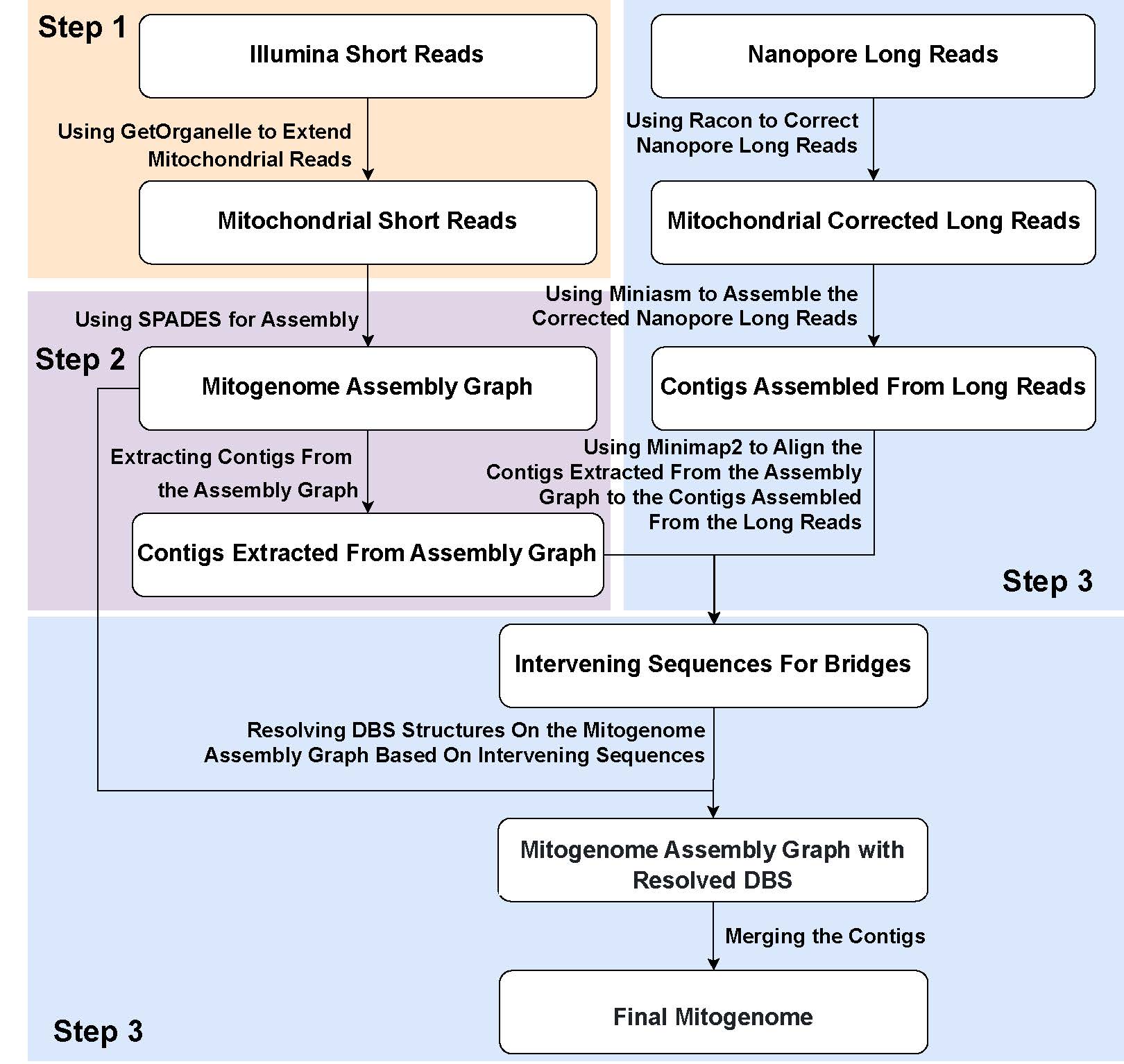

Supplement: Supplementary file 1 [file Image_1.jpeg]

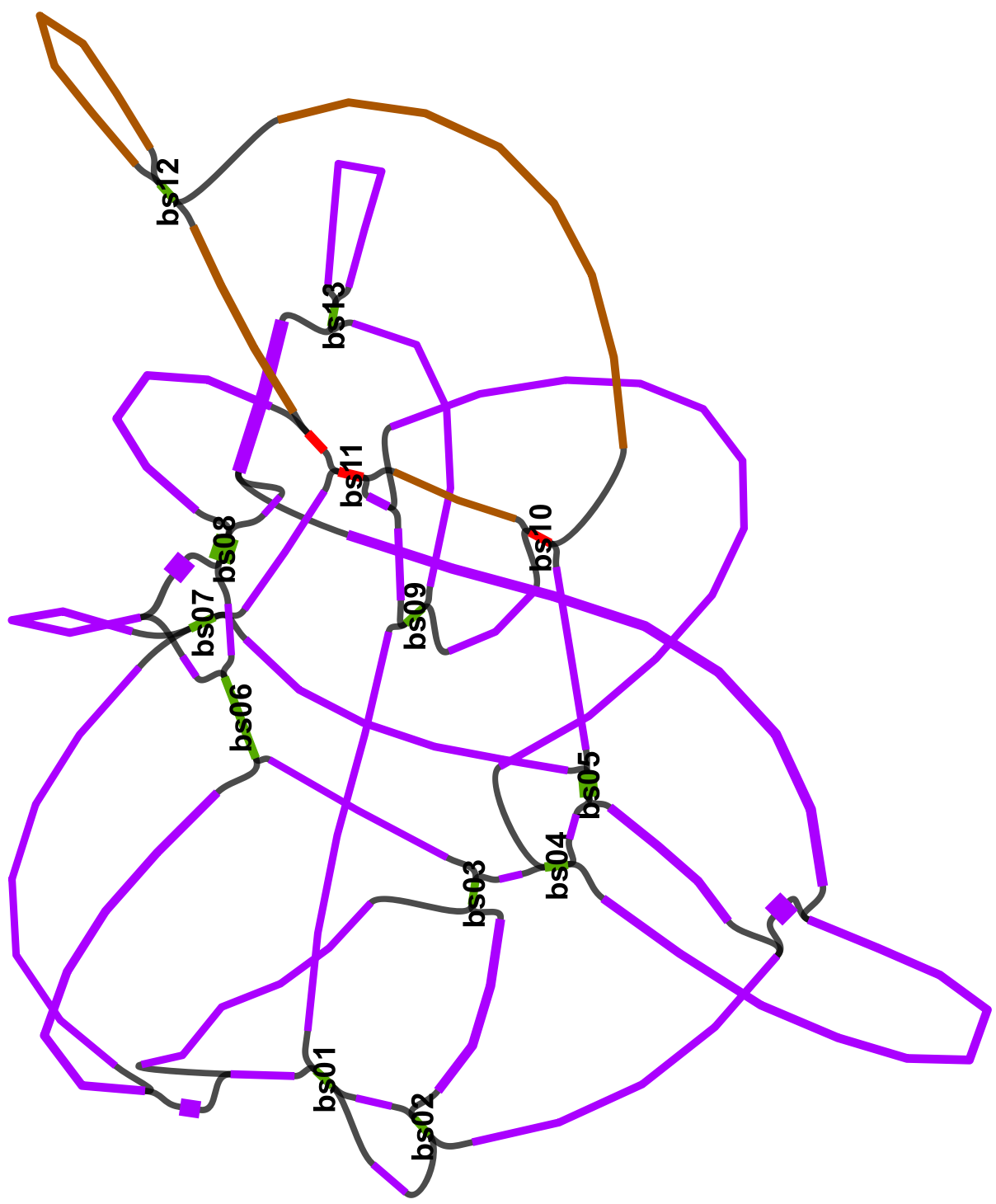

Supplement: Supplementary file 2 [file Image_2.png]

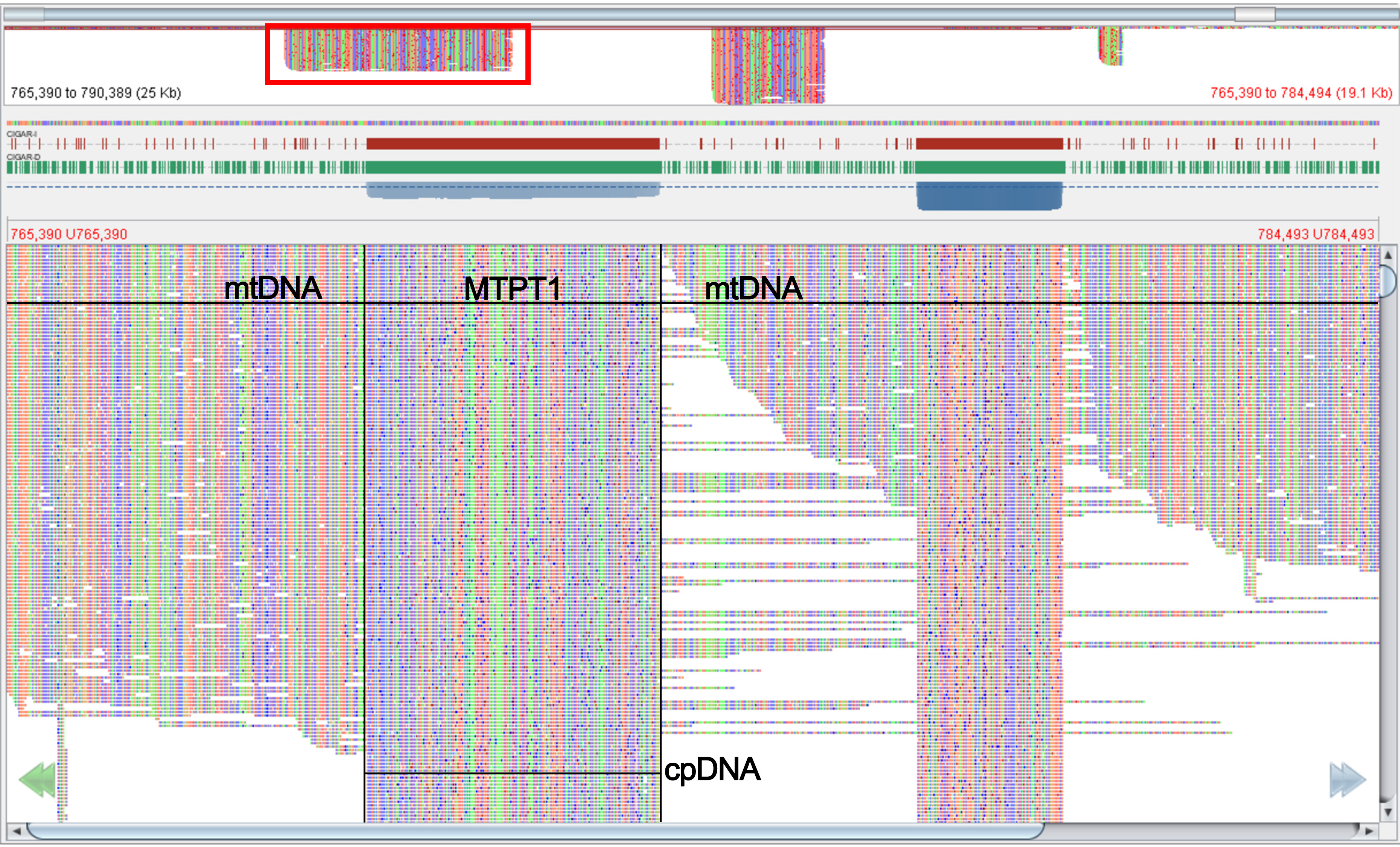

Supplement: Supplementary file 3 [file Image_3.jpeg]

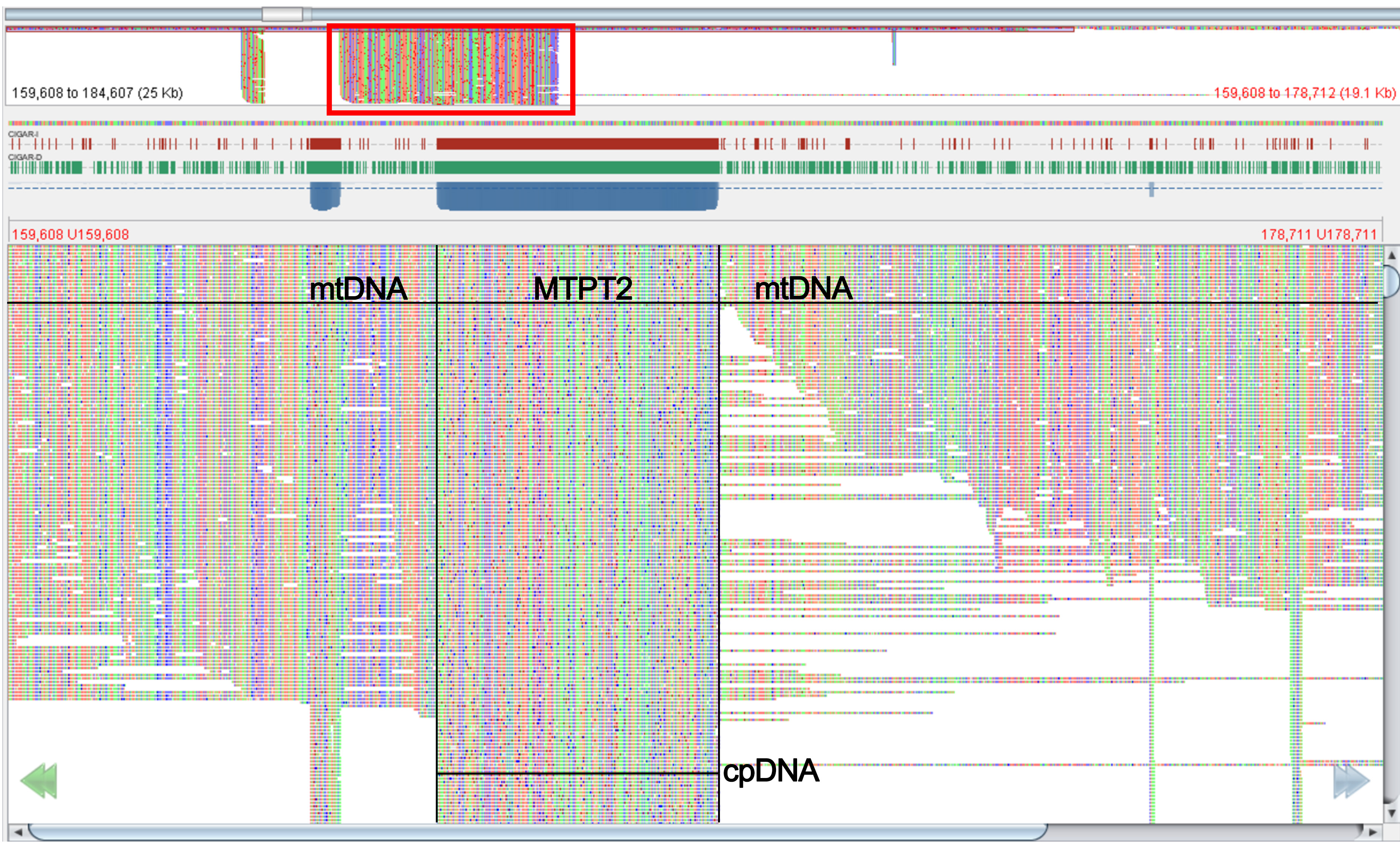

Supplement: Supplementary file 4 [file Image_4.jpeg]

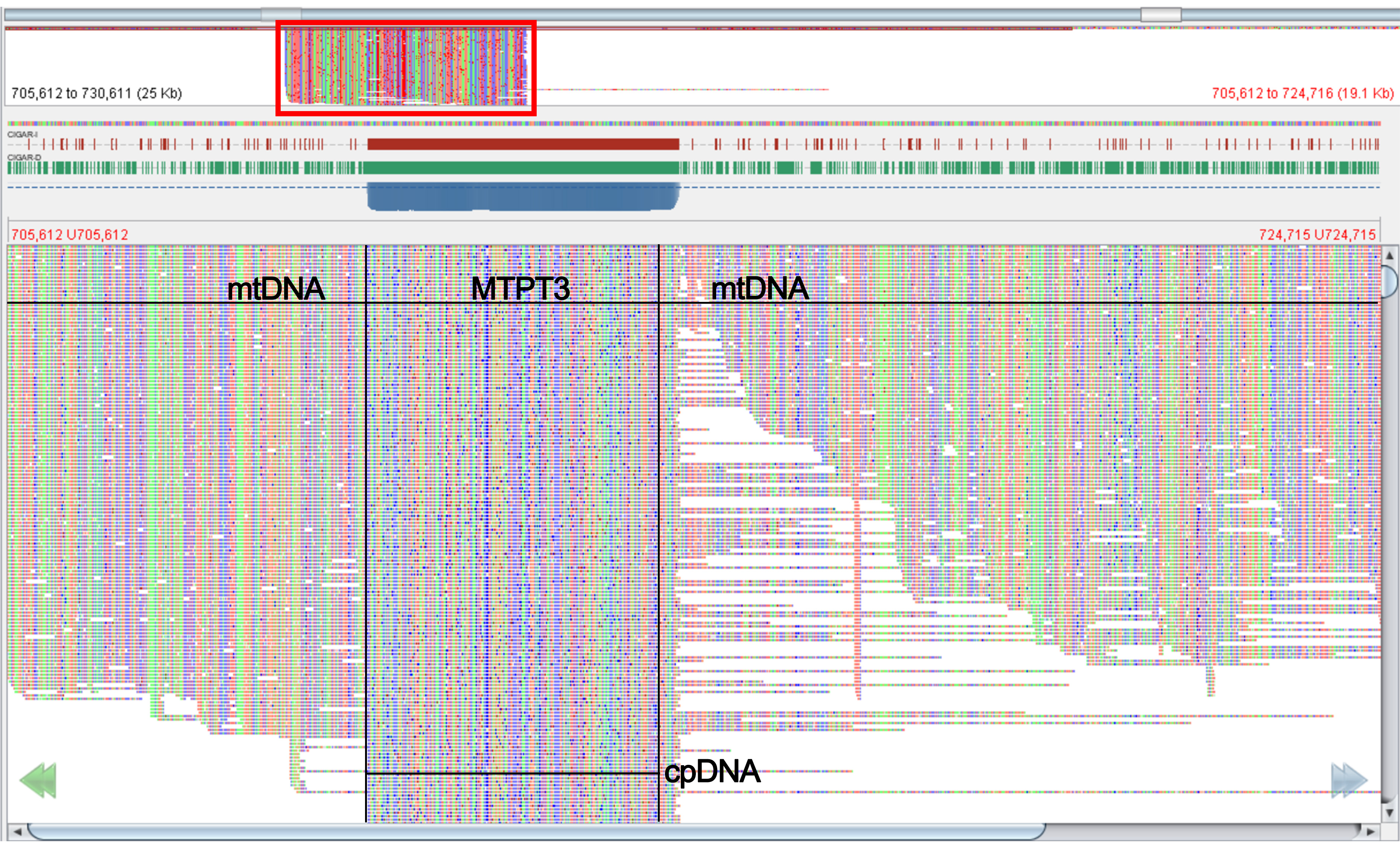

Supplement: Supplementary file 5 [file Image_5.jpeg]

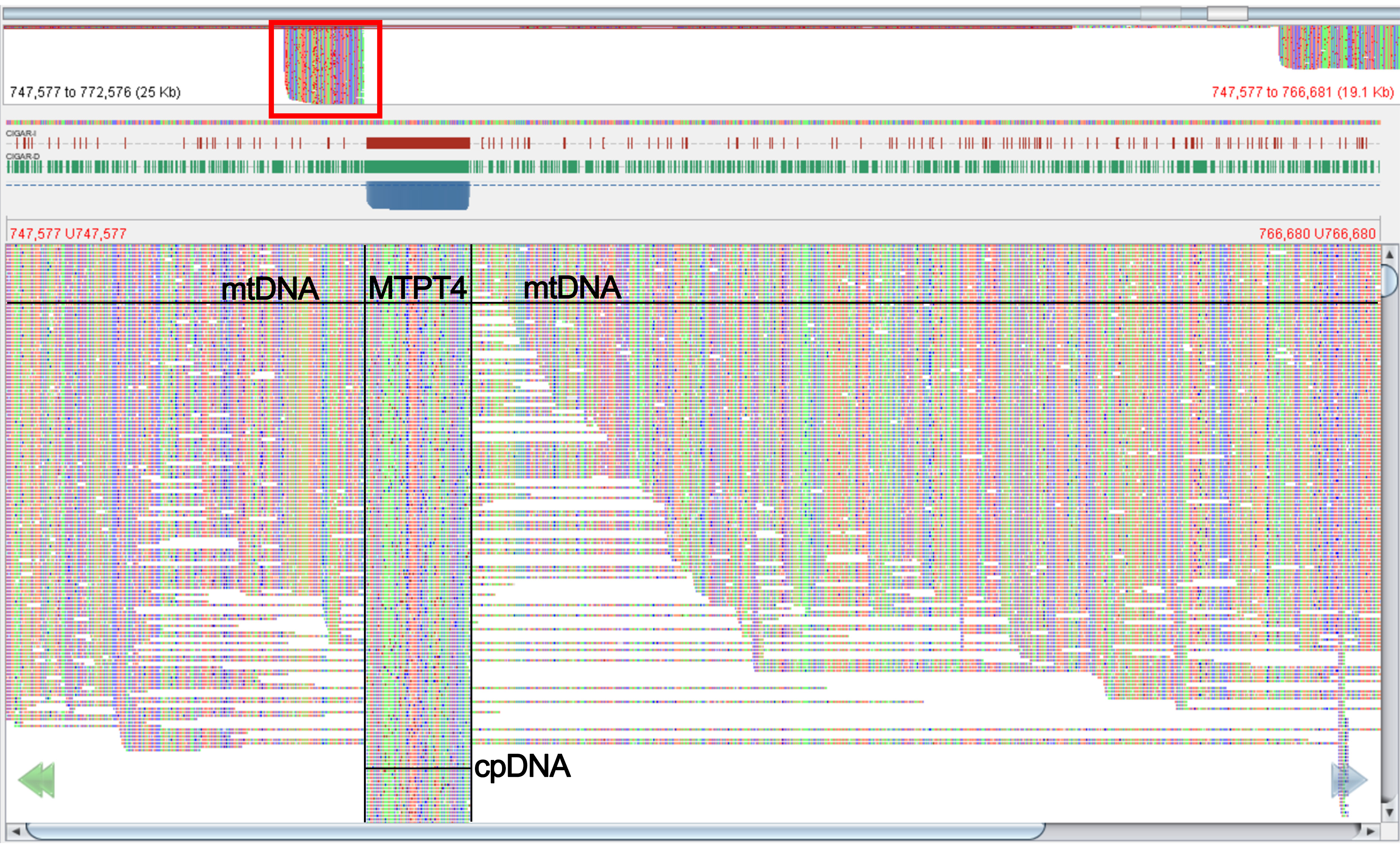

Supplement: Supplementary file 6 [file Image_6.jpeg]

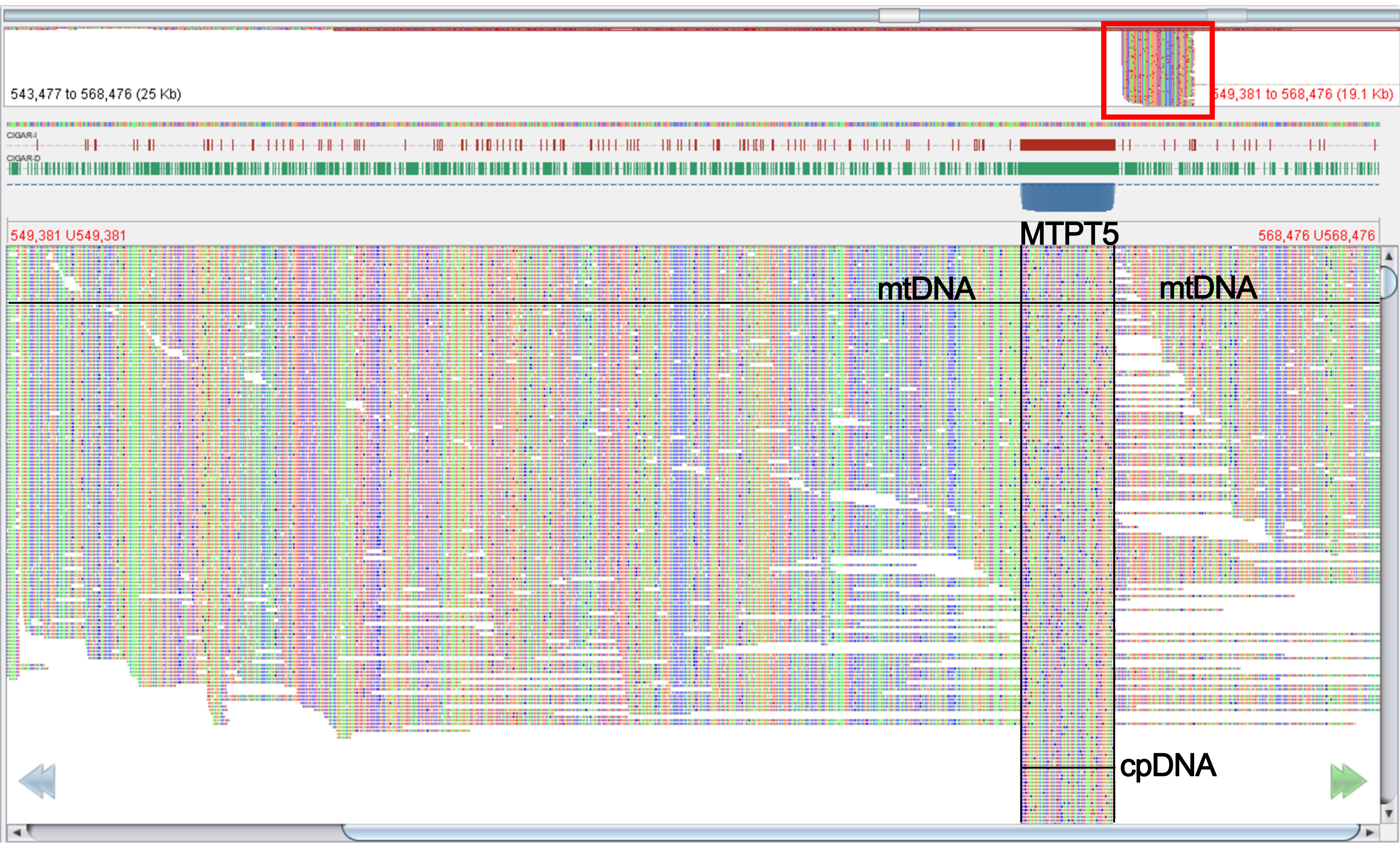

Supplement: Supplementary file 7 [file Image_7.jpeg]

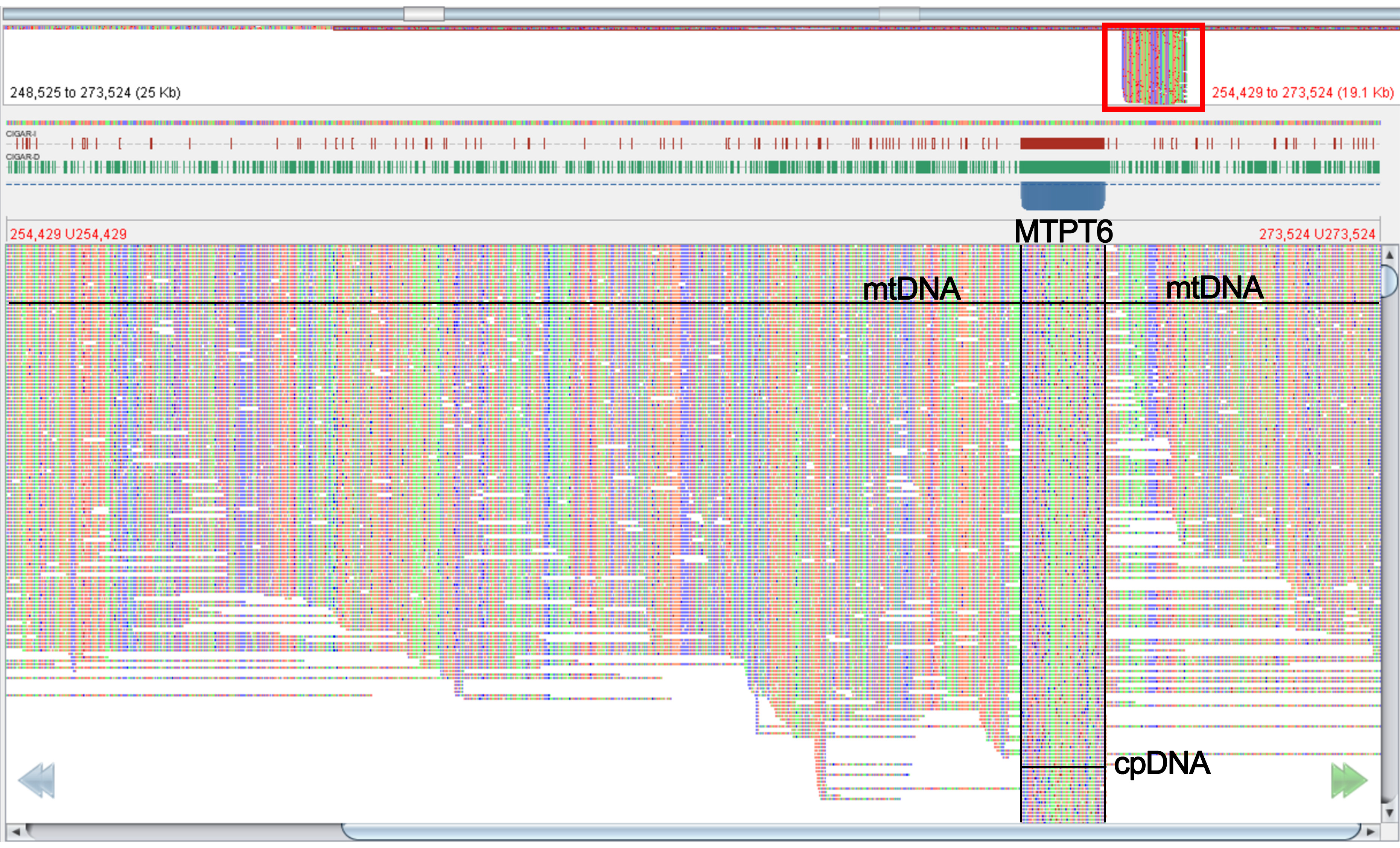

Supplement: Supplementary file 8 [file Image_8.jpeg]

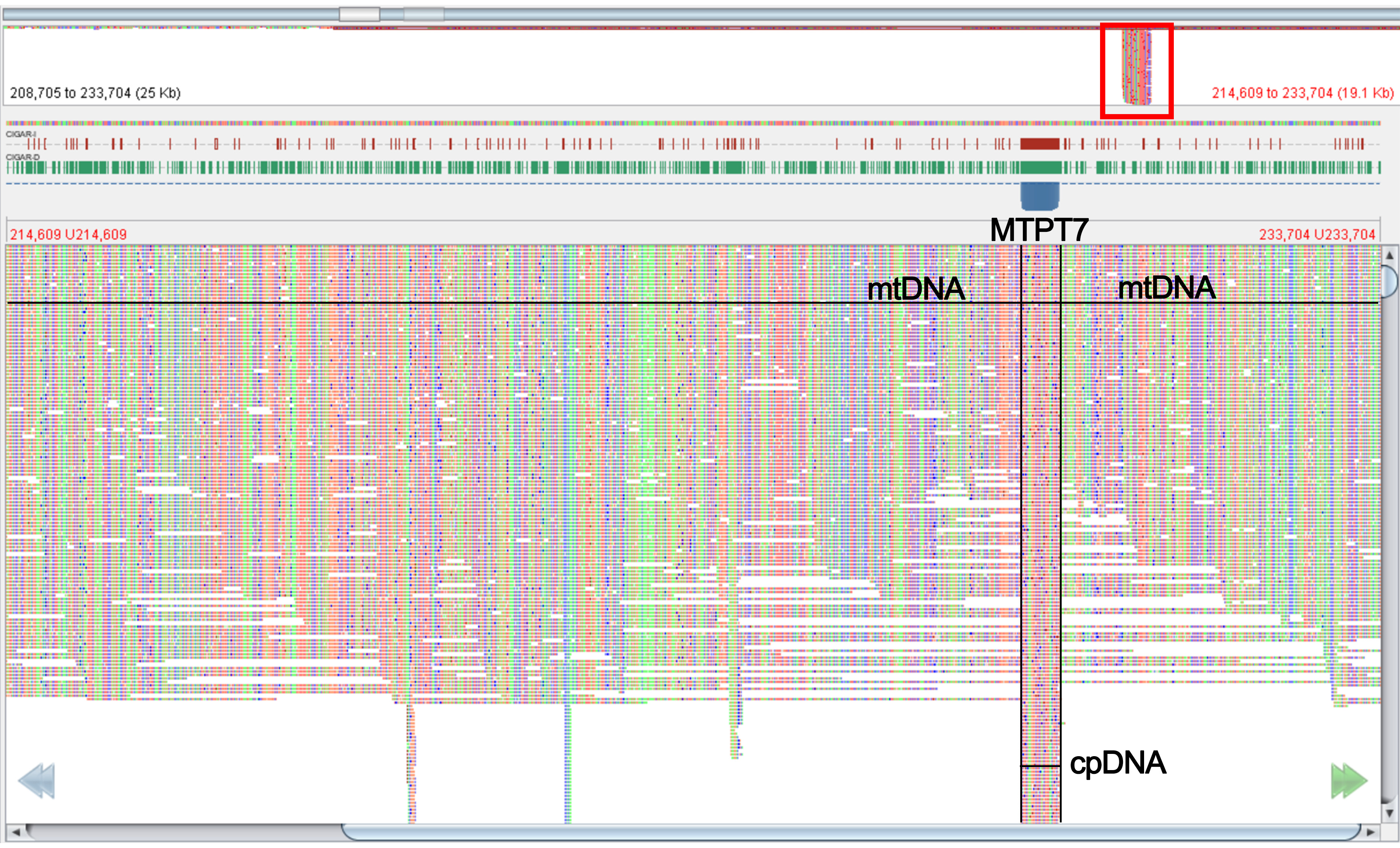

Supplement: Supplementary file 9 [file Image_9.jpeg]

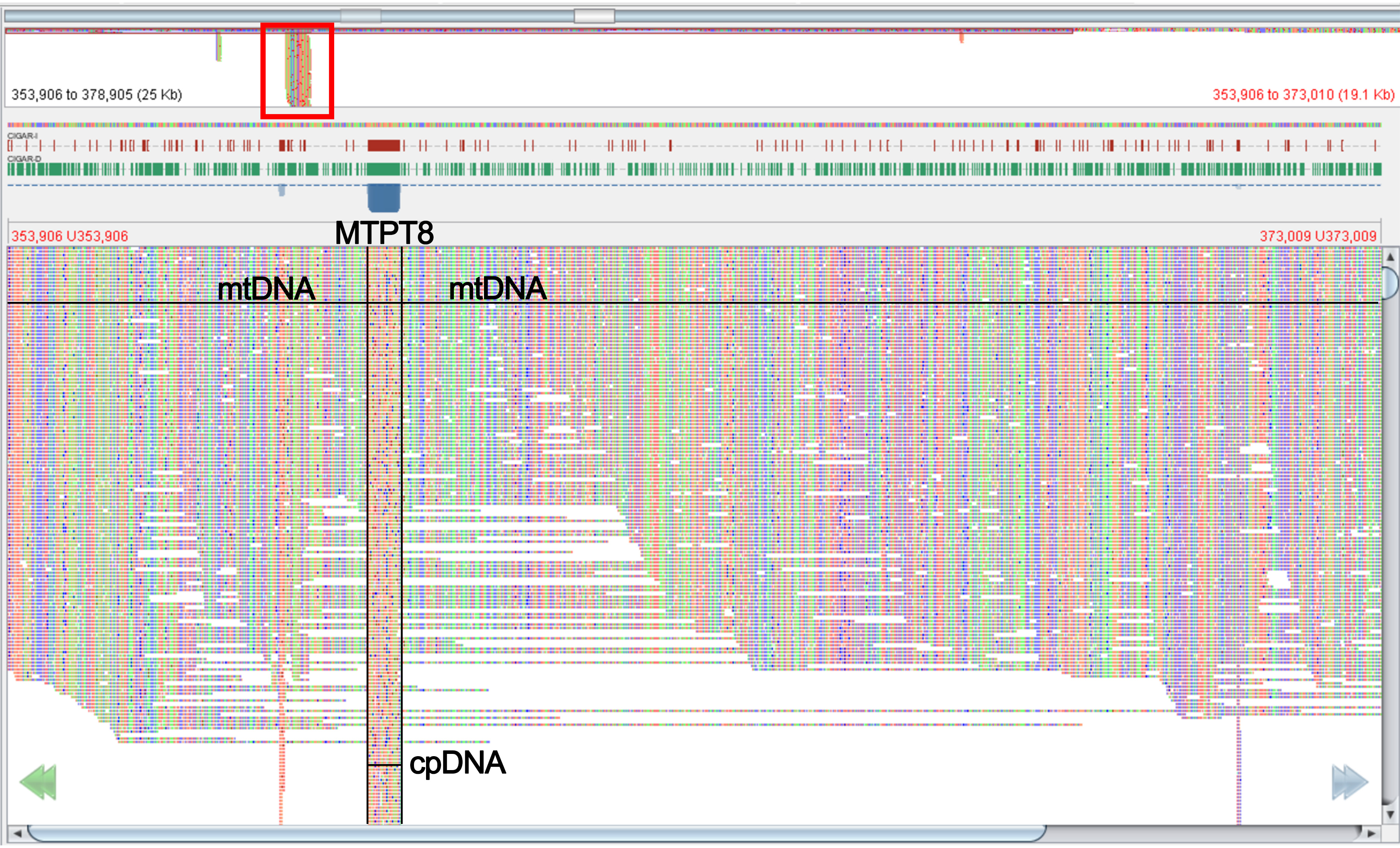

Supplement: Supplementary file 10 [file Image_10.jpeg]

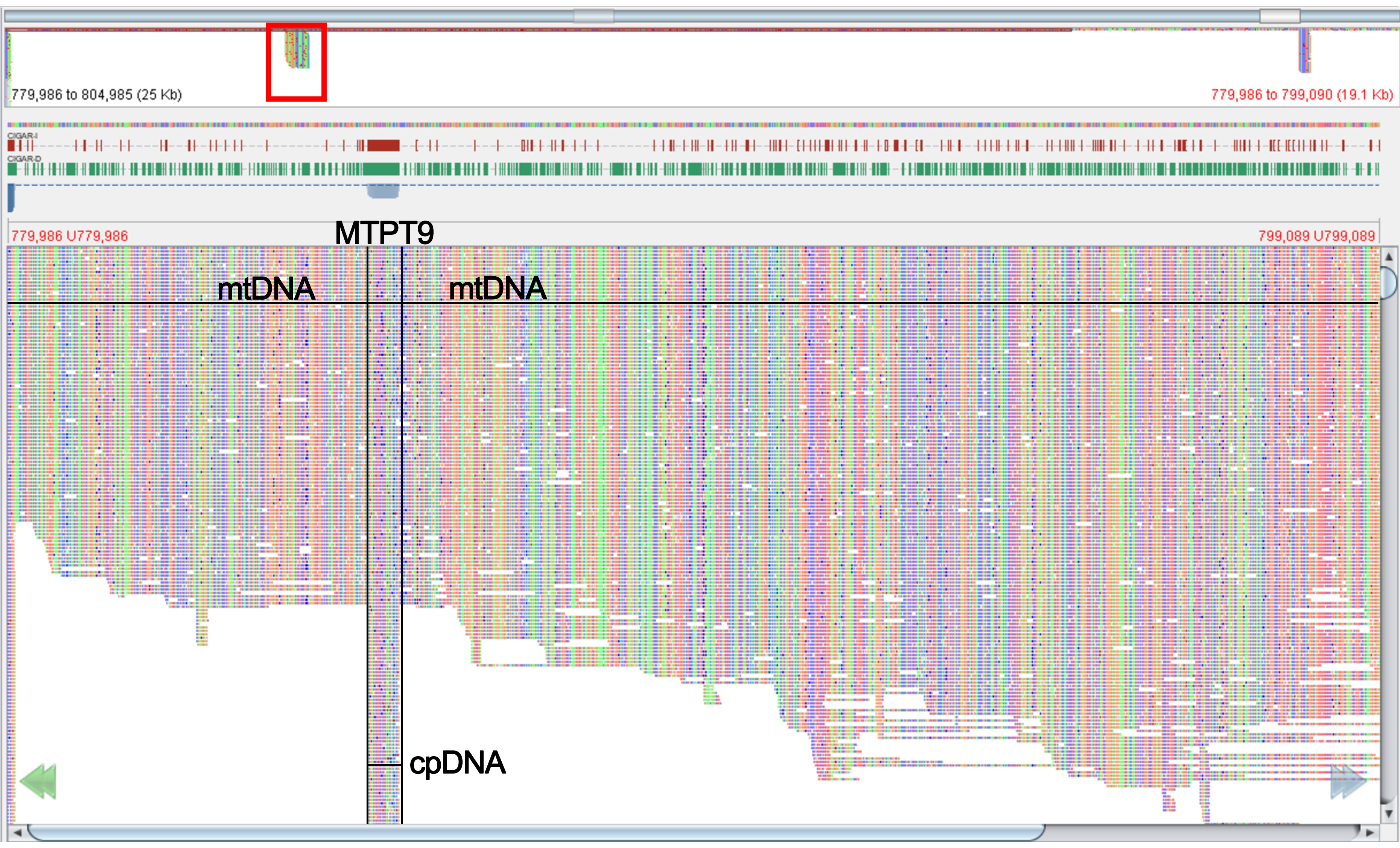

Supplement: Supplementary file 11 [file Image_11.jpeg]

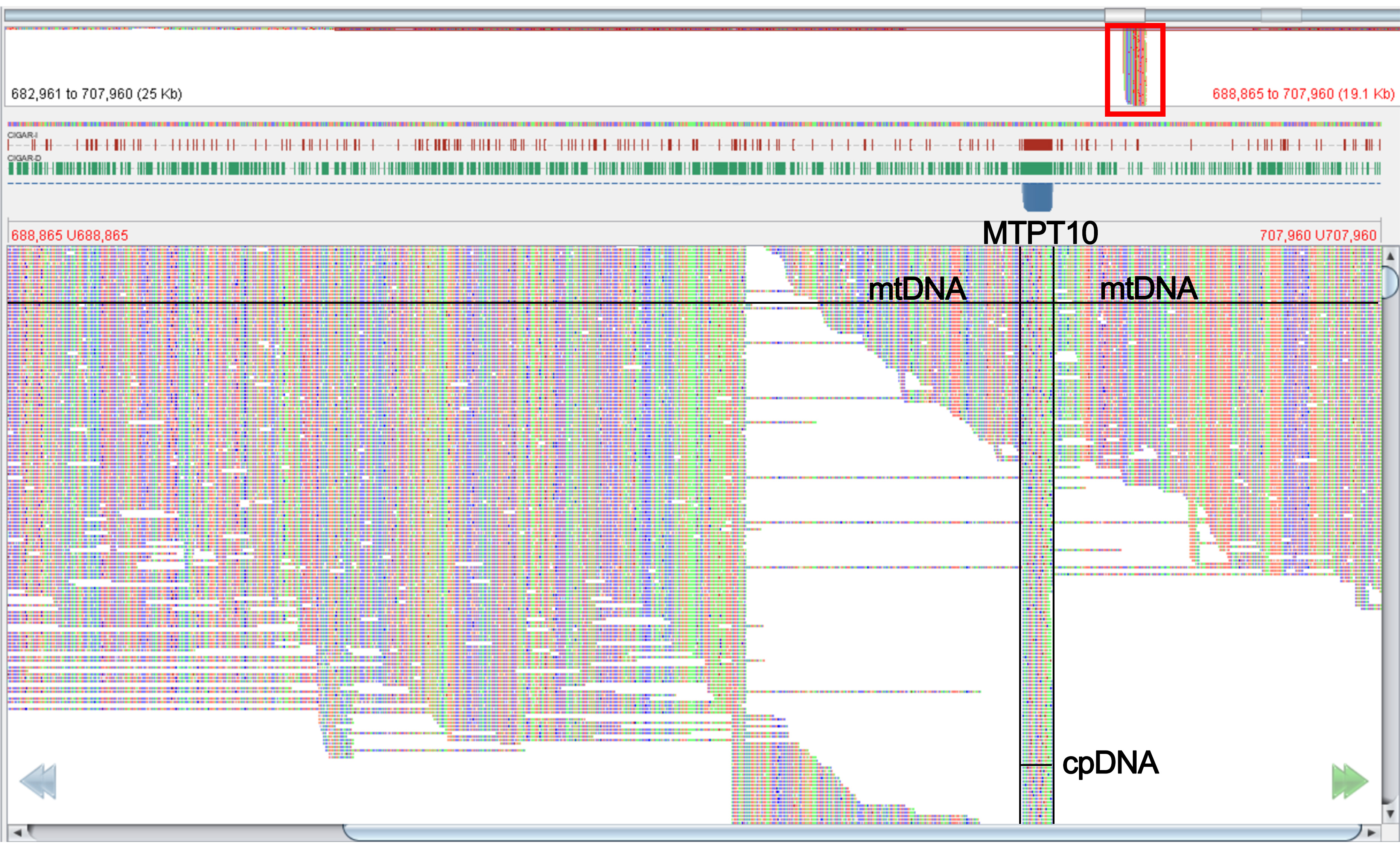

Supplement: Supplementary file 12 [file Image_12.jpeg]

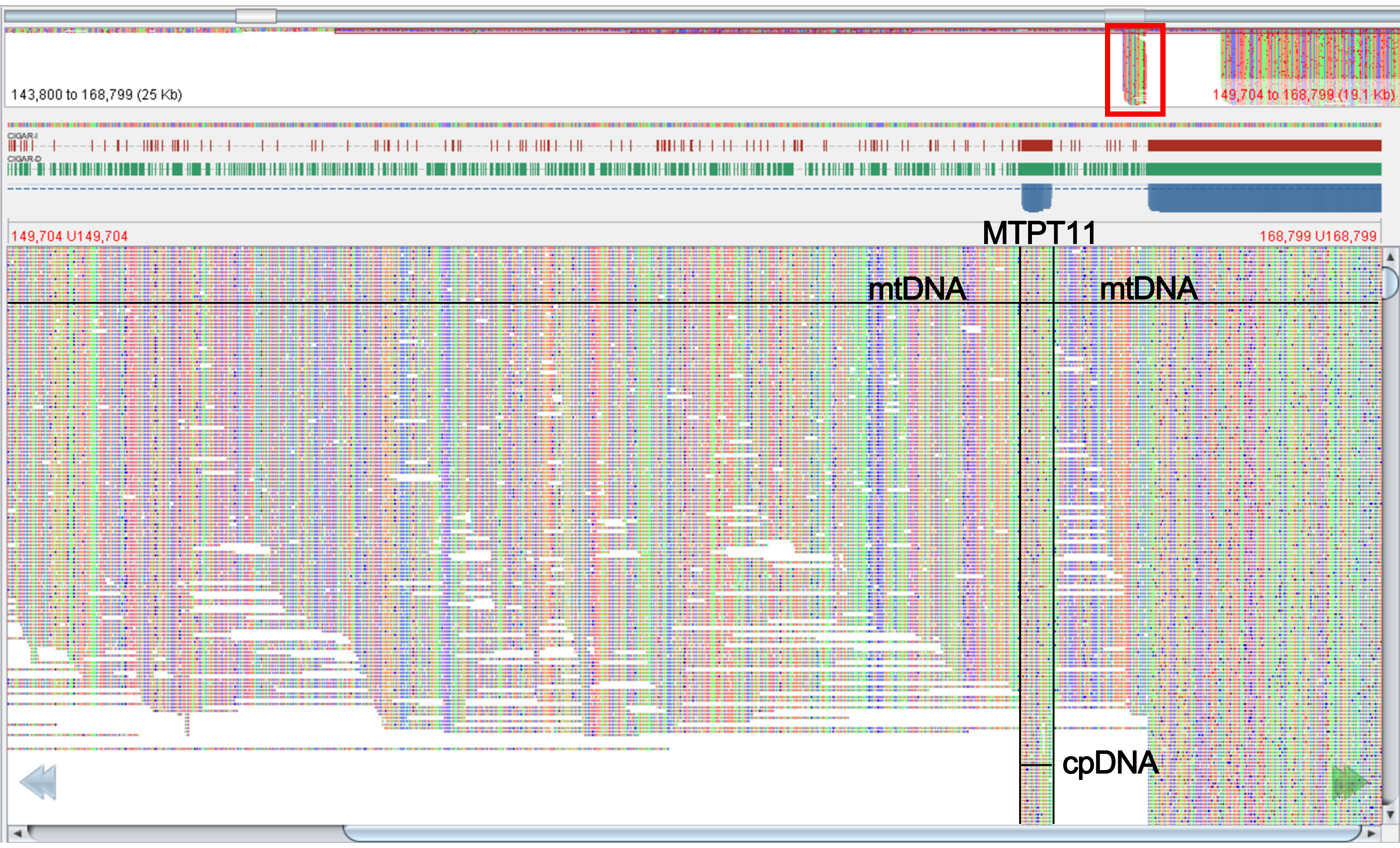

Supplement: Supplementary file 13 [file Image_13.jpeg]

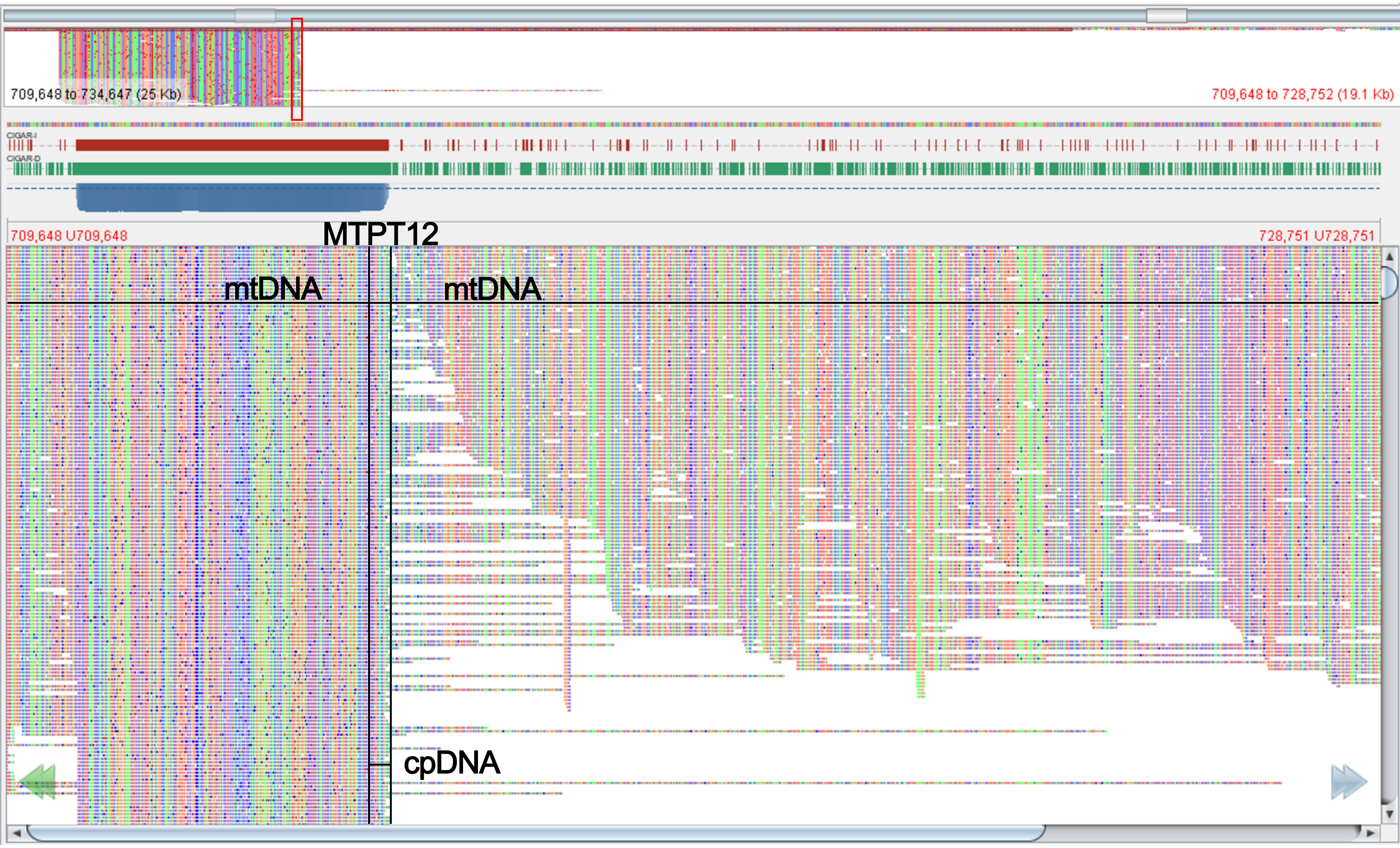

Supplement: Supplementary file 14 [file Image_14.jpeg]

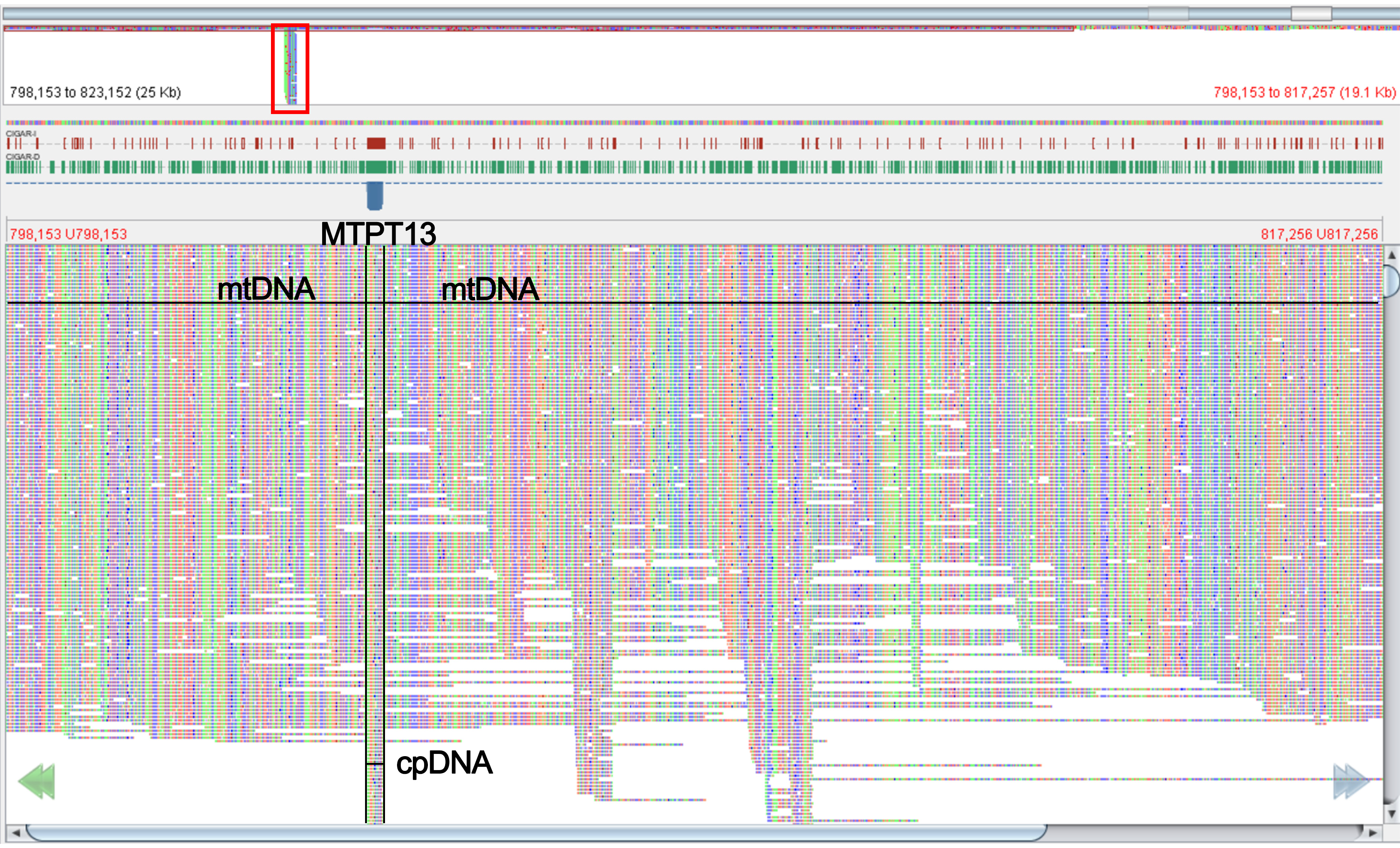

Supplement: Supplementary file 15 [file Image_15.jpeg]

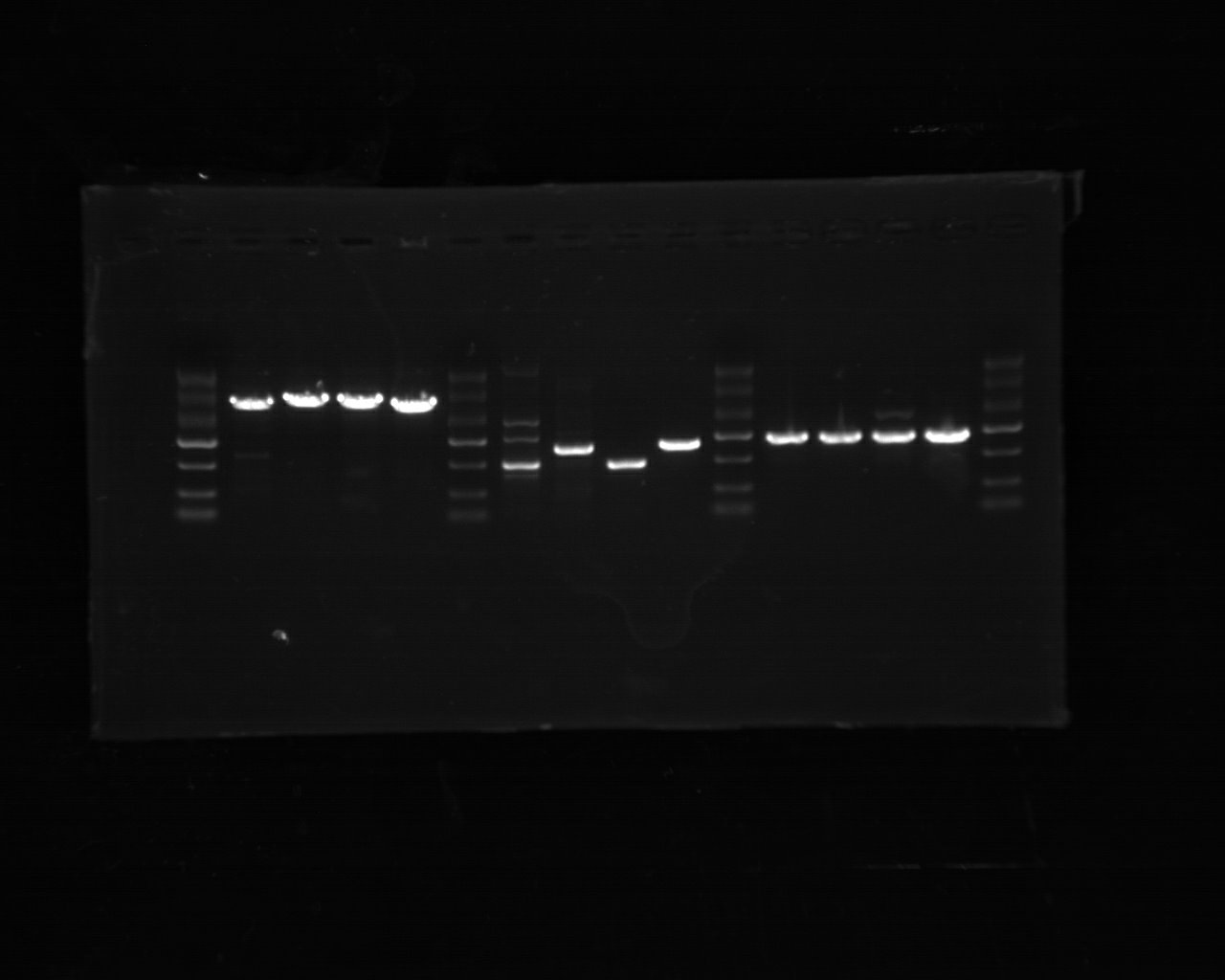

Supplement: Supplementary file 17 [file DataSheet_1.zip › Supplementary File 5.jpg]
